# Real-world pharmacovigilance analysis of pralatrexate using the FDA adverse event reporting system database

**DOI:** 10.3389/fphar.2026.1773445

**Published:** 2026-04-08

**Authors:** Wei Yang, Qing Wu, Yu Zheng, Wanyan Tang, Zonglang Lai, Shuang Fei, Weiqi Nian

**Affiliations:** 1 Department of Oncology, Chongqing Hospital of Traditional Chinese Medicine, Chongqing, China; 2 Chongqing Municipal Health Commission Key Laboratory for Translational Research in Integrated Chinese and Western Medicine Oncology Diagnosis and Treatment, Chongqing, China; 3 Department of Oncology, Daping Hospital, Army Medical University, Chongqing, China

**Keywords:** adverse events, FDA adverse event reporting system, peripheral T cell lymphoma, pharmacovigilance, pralatrexate

## Abstract

**Introduction:**

Pralatrexate, the first United States Food and Drug Administration (FDA)-approved selective antifolate agent for relapsed/refractory peripheral T cell lymphoma (PTCL), has an incompletely defined postmarketing safety profile. In this study, we systematically assessed the characteristic spectrum of pralatrexate-related adverse events (AEs) by integrating FDA Adverse Event Reporting System (FAERS) data to improve the safety evidence for this drug and provide scientific support for optimizing clinical risk management strategies.

**Methods:**

A primary suspected drug-related AE analysis set was constructed following a standardized data cleaning process based on all AE reports from the FAERS database from the first quarter of 2004 through the first quarter of 2025. Signal detection analysis was performed using four algorithms, including the reporting odds ratio, proportional reporting ratio, Bayesian confidence propagation neural network, and multi-item gamma Poisson shrinker algorithms. Subgroup analyses were performed for sex, age, and report type categories to explore differences.

**Results:**

A total of 2,241 AE reports were extracted from 563 patients using pralatrexate. Patients were predominantly male (58.44%) and aged ≥65 years (45.65%). AEs primarily affected the gastrointestinal, hematopoietic, and integumentary systems. “General disorders and administration site conditions” was the most frequently reported system organ class (N = 354, 15.80%). We detected 84 positive signals, including expected AEs such as mucositis, myelosuppression, skin reaction, and abnormal liver function, as well as several potential new risk signals including hypochloremia, increased platelet counts, prolonged activated partial thromboplastin time, and intestinal ischemia. Mucositis and hematologic toxicity were the primary factors leading to severe outcomes such as death, life-threatening conditions, and hospitalization. The median time to onset of pralatrexate AEs was 16 days (interquartile range, 6–59 days), and the Weibull distribution test was consistent with the early failure type. Subgroup analyses showed that the safety profile of pralatrexate was generally consistent between sexes, but risk of toxic epidermal necrolysis was significantly higher in female patients (reporting odds ratio = 5.14, 95% confidence interval: 1.06–24.79). In addition, AEs showed a significant age-related relationship, with mucosal injury and metabolic disorders predominating in patients aged ≥65 years, whereas malignant neoplasm progression and abnormal liver function were more common in those aged <65 years.

**Conclusion:**

This study elucidated the safety profile of pralatrexate in real-world settings, validating its established adverse reactions and identifying novel potential AEs. Our findings suggest that clinicians should implement personalized monitoring on the basis of patient sex and age, with a particular emphasis on mucosal toxicity and metabolism-related AEs in patients ≥65 years old. Furthermore, the adverse reaction spectrum of pralatrexate may be broader than previously recognized, necessitating further investigation to optimize medication safety management.

## 1 Introduction

Peripheral T cell lymphoma (PTCL) is a highly aggressive lymphoma that accounts for approximately 10%–15% of newly diagnosed cases of non-Hodgkin’s lymphoma (Vose et al., 2008). Owing to the insidious onset of the disease, the majority of patients are diagnosed at an advanced stage, and the prognosis remains poor even after stem cell transplantation (Rodriguez et al., 2009; Chang et al., 2024). PTCL responds poorly to conventional chemotherapy and is prone to developing drug resistance (O'Connor et al., 2011). Patients with relapsed or refractory disease have limited salvage therapy options, contributing to the extremely poor prognosis. Therefore, novel therapeutic strategies are urgently needed. In 2009, the United States Food and Drug Administration (FDA) granted accelerated approval for pralatrexate for the treatment of individuals with relapsed/refractory PTCL, making it the first drug approved for this indication. Since then, pralatrexate has been approved for the same indication in multiple countries and regions, including the European Union, Canada, Australia, Switzerland, a majority of countries in the Middle East, Japan, and China.

Pralatrexate (10-propynyl-10-deazaminopterin) is a folate analog that competitively inhibits dihydrofolate reductase (DHFR), leading to thymidine depletion, which, in turn, causes DNA replication errors and induces tumor cell apoptosis (Malik et al., 2010). The use of antifolate medications is often accompanied by a high incidence of serious adverse reactions. Clinical trial data showed that approximately 23% of patients discontinued pralatrexate treatment owing to adverse drug reactions, and a considerable proportion of patients required dosage adjustments (O'Connor et al., 2011). Common adverse reactions include mucositis, thrombocytopenia, anemia, neutropenia, fever, epistaxis, and liver function abnormalities. In addition, the drug carries the risk of causing severe and even fatal skin reactions, and other potentially life-threatening toxic reactions (U.S. Food and Drug Administration, 2020).

With the approval of pralatrexate for marketing, its clinical application has continued to grow, and understanding of its pharmacological properties, treatment strategies, and dosing regimens has been steadily refined. The new strategies appear to contribute to improved patient tolerance, particularly by reducing the risk of toxic reactions such as stomatitis (O'Connor et al., 2017). However, the currently available clinical trial data remain relatively limited, and evidence of the real-world safety profile of pralatrexate is still insufficient. In this context, we utilized data from the FDA Adverse Event Reporting System (FAERS) to address two core questions: First, in the real-world setting, which AEs are most commonly associated with pralatrexate, and what is the likelihood of developing these AEs? Second, how does the pralatrexate safety profile compare with existing safety data? Through in-depth analysis of these questions, this research aims to provide critical insights into the safe clinical use and risk management of this drug.

The FAERS is an open-access public database established by the United States FDA to support postmarketing safety surveillance of all approved drugs and therapeutic biological products. It incorporates adverse drug events and medication error reports collated by the FDA, which, to some extent, reflect the safety profiles of medications in real-world settings and aid in identifying potential risk signals that may be difficult to detect in clinical trials (Xu, 2015). To extend the existing safety information on pralatrexate, which primarily stems from clinical trials, this study utilizes the FAERS database and disproportionality analysis methods to systematically evaluate the safety profile of pralatrexate, with the aim of providing more comprehensive and clinically relevant evidence for guiding rational drug use.

## 2 Materials and methods

### 2.1 Data sources

The raw data were sourced from the FAERS, which can be accessed at https://fis.fda.gov/extensions/FPD-QDE-FAERS/FPD-QDE-FAERS.html. The FAERS database report contains data from seven datasets: demographic and administrative information (DEMO), drug information (DRUG), adverse drug reaction information (REAC), patient outcome information (OUTC), reporting source information (RPSR), date of treatment initiation and end date of reported medication (THER), and medication administration indications (INDI). For this study, we extracted all raw ASCII data spanning 85 quarters from the first quarter of 2004 to the first quarter of 2025, and subsequently performed statistical analysis using SAS 9.4.

### 2.2 Data processing and target population screening

#### 2.2.1 Data deduplication

Duplicate removal was performed according to the rules specified on page 11 of the ASC_NTS.DOC file (Supplementary Material 1) from the first quarter of the 2004 FAERS ASCII dataset. We selected the primary identifiers (PRIMARYID), case identifier (CASEID), and FDA receipt date (FDA_DT) fields from the DEMO table (Supplementary Material 2). The records were sorted by CASEID, FDA_DT, and PRIMARYID. Among reports with the same CASEID, the report with the latest FDA_DT was retained. For reports with both the same CASEID and FDA_DT, the report with the largest PRIMARYID value was retained. An example of a deduplicated report is provided in Supplementary Table S1 (Supplementary Material 3).

#### 2.2.2 Report removal

Since the first quarter of 2019, each quarterly dataset has included a new text file that lists deleted files. After data deduplication, reports with CASEIDs specified in this list must be removed. This rule is detailed on page 10 of the FAQS.PDF file (Supplementary Material 4) from the first quarter of 2019 ASCII dataset.

#### 2.2.3 Medical dictionary for regulatory activities preferred term standardization

The FAERS database utilizes preferred terms (PTs) from the Medical Dictionary for Regulatory Activities (MedDRA) to describe AEs. MedDRA is updated twice each year, in March and September, which may involve modifications to PTs and corresponding system organ class (SOC) classifications. To standardize the PTs for this study, we extracted MedDRA version 28.0 SOCs and PTs for subsequent analysis.

#### 2.2.4 Target population screening

In the database, each report contains a unique primary suspected (PS) drug. We considered only the PS drug when developing our target patient population. Cases for which the PS drug was “pralatrexate” were classified as the target drug population, while others were assigned to the non-target drug population. All drug names were standardized using the WHO Drug Dictionary (March 2025 release), with subsequent screening and analyses conducted exclusively using the standardized generic names.

### 2.3 Signal detection

This study employed the disproportionality analysis method to detect signals for pralatrexate-associated AEs; the specific algorithms are detailed in Supplementary Table S2 (Supplementary Material 3). To mitigate potential biases inherent to single methods and enhance the stability and reliability of the results, four distinct algorithms were simultaneously applied for signal detection, including the reporting odds ratio (ROR), proportional reporting ratio (PRR; using the comprehensive criteria established by the UK Medicines and Healthcare products Regulatory Agency for signal determination), Bayesian confidence propagation neural network (BCPNN), and multi-item gamma Poisson shrinker (MGPS) algorithms. A positive signal (i.e., a potential risk signal) was identified only when all four methods met the threshold. The calculation formulas and specific thresholds for each algorithm are provided in Supplementary Table S3 (Supplementary Material 3). Additionally, to identify sex-, age-, and report type-based disproportionate signals following pralatrexate administration, the ROR method was used. We assessed sex-based risk differences using published criteria (Bate and Evans, 2009), by which an ROR >1 with a 95% confidence interval (CI) lower limit >1 suggests that female patients are more likely to report a specific AE than male patients. Conversely, an ROR <1 with a 95% CI upper limit <1 indicates that male patients are more likely to report a specific AE than female patients.

In this study, the term “signal” is used as a professional pharmacovigilance term, specifically referring to an association identified through disproportionality analysis that suggests a statistically disproportionate report between the target drug and a specific AE. It represents a potential association hypothesis that requires further pharmacological and clinical validation, rather than a confirmed causal relationship. To emphasize this nature, it is also referred to as a “disproportionality signal” or a “potential signal” in the following sections.

### 2.4 Time to onset analysis

The time to onset of pralatrexate-associated AEs was calculated as the interval between the date of treatment initiation and the date of AE onset. The median and interquartile range were employed to describe the duration of the events, excluding reports with incomplete date information or AEs occurring prior to therapy initiation.

## 3 Results

### 3.1 Descriptive analysis

**FIGURE 1:**
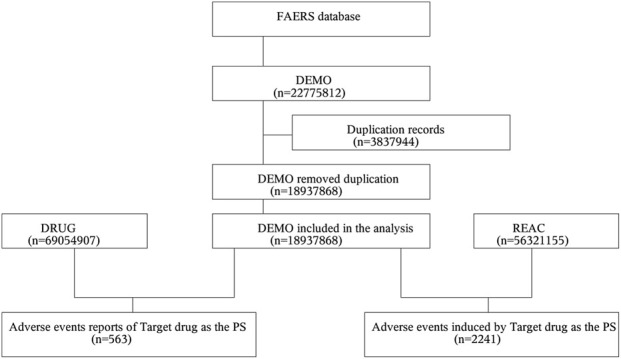
Data screening flow diagram for adverse events associated with pralatrexate in the FDA Adverse Event Reporting System database. Abbreviation: DEMO, Demographics; PS, Primary suspect; REAC, Reactions.

From the first quarter of 2004 to the first quarter of 2025, the FAERS database contained 56,321,155 AE reports involving 18,937,868 patients. Following standardized screening (Figure 1), 2,241 AE instances associated with pralatrexate were identified across 563 patient reports; the detailed case characteristics are presented in Table 1. Demographic analysis revealed a predominance of male patients (58.44%) and those aged ≥65 years (45.65%) in pralatrexate-related cases. The year with the highest proportion of reports (17.41%) was 2021. Regarding reporting sources, 44.76% of reports originated from physicians. Cases clustered predominantly in the United States of America (41.56%) and Japan (32.15%), with the United Kingdom (9.59%), Colombia (6.57%), and Bulgaria (3.37%) completing the top five countries. The most frequent clinical outcome was hospitalization (43.16%), and 34.10% of these AEs were reported within 30 days post-administration.

**TABLE 1:** Characteristics of AE reports related to pralatrexate.

Characteristics	Case number (case proportion, %)
Sex	
Female	200 (35.52)
Male	329 (58.44)
Not specified	34 (6.04)
Age (years)	
<18	0 (0.00)
18–44	52 (9.24)
45–64	149 (26.47)
≥65	257 (45.65)
Not specified	105 (18.65)
Report year	
2009	6 (1.07)
2010	54 (9.59)
2011	41 (7.28)
2012	36 (6.39)
2013	44 (7.82)
2014	3 (0.53)
2015	25 (4.44)
2016	20 (3.55)
2017	13 (2.31)
2018	44 (7.82)
2019	39 (6.93)
2020	37 (6.57)
2021	98 (17.41)
2022	53 (9.41)
2023	35 (6.22)
2024	9 (1.60)
2025	6 (1.07)
Reporter	
Consumer	75 (13.32)
Not specified	21 (3.73)
Other health-professional	138 (24.51)
Pharmacist	77 (13.68)
Physician	252 (44.76)
Reported countries (top five)	
United States Of America	234 (41.56)
Japan	181 (32.15)
United Kingdom	54 (9.59)
Colombia	37 (6.57)
Bulgaria	19 (3.37)
Outcomes^a^	
Life-threatening	58 (10.30)
Hospitalization - initial or prolonged	243 (43.16)
Disability	10 (1.78)
Death	145 (25.75)
Congenital anomaly	0 (0.00)
Required intervention to prevent permanent impairment/Damage	1 (0.18)
Other	324 (57.55)
Adverse event occurrence time (days)	
0-30 d	192 (34.10)
31-60 d	49 (8.70)
61-90 d	21 (3.73)
91-120 d	12 (2.13)
121-150 d	8 (1.42)
151-180 d	7 (1.24)
181-360 d	14 (2.49)
>360 d	13 (2.31)
Missing or outlier (<0)	247 (43.87)

^a^ 1. Outcomes were analyzed at the patient level rather than per individual adverse event. Since a single patient may have multiple outcomes in the database, the sum of outcome percentages may exceed 100%. 2. For detailed annual stratified data regarding outcomes, please refer to Supplementary Figure S1 in Supplementary Material 3.

### 3.2 AE classifications at the SOC level

**FIGURE 2:**
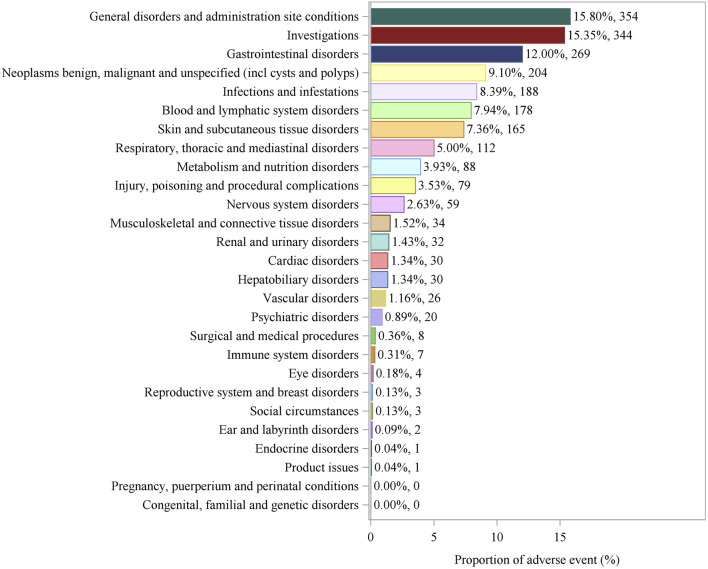
System organ class distribution of adverse events associated with pralatrexate.

Based on the SOC analysis (Figure 2), the 2,241 AEs associated with pralatrexate involved 25 organ systems. The primary SOC terms that accounted for >5% of AEs included seven categories: “general disorders and administration site conditions” (15.80%); “investigations” (15.35%); “gastrointestinal disorders” (12.00%); “neoplasms benign, malignant and unspecified (incl cysts and polyps)” (9.10%); and “infections and infestations” (8.39%). The secondary SOC terms (accounting for 1%–5% of AEs) comprised nine categories; “respiratory, thoracic, and mediastinal disorders” (5.00%); “metabolism and nutrition disorders” (3.93%); “injury, poisoning, and procedural complications” (3.53%); “nervous system disorders” (2.63%); and “musculoskeletal and connective tissue disorders” (1.52%) represented the predominant categories. The remaining AEs involved other organ systems, such as the urinary, hepatobiliary, and cardiac systems. Rare categories (accounting for <1%) spanned nine categories, including “psychiatric disorders” (0.89%), “surgical and medical procedures” (0.36%), and “immune system disorders” (0.31%), among which “endocrine disorders” (0.04%) and “product issues” (0.04%) were reported with the lowest frequency.

### 3.3 Disproportionality analysis at the PT level

**FIGURE 3:**
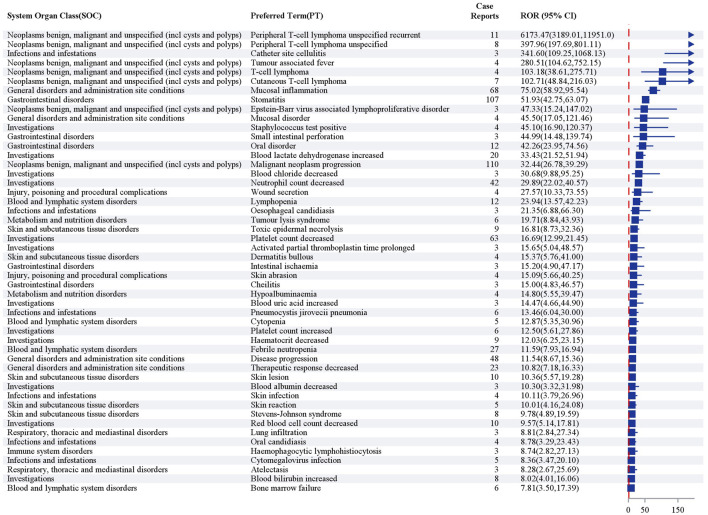
Top 50 preferred terms by reporting odds ratio value after screening for positive signals by reporting odds ratio, proportional reporting ratio, Bayesian confidence propagation neural network, and multi-item gamma Poisson shrinker algorithms.

Using four disproportionality analysis algorithms (ROR, PRR, BCPNN, and MGPS), we detected 84 pralatrexate-associated AE signals at the PT level in the FAERS database. All signals were systematically categorized by expectedness using the pralatrexate summary of product characteristics (SmPCs), patients’ primary disease, and published literature (Supplementary Table S4 in Supplementary Material 3). These signals were ranked by descending ROR value; Figure 3 presents the 50 PTs with the strongest signal intensities. The analysis revealed that the majority of positive signals were expected AEs, highly consistent with the documentation in the pralatrexate SmPC, including hematologic toxicities (e.g., neutropenia, thrombocytopenia, and anemia), dermatologic toxicities (e.g., skin reactions), and other significant AEs (e.g., mucositis, tumor lysis syndrome, and abnormal liver function). The study also identified a positive signal reported in the literature but not yet included in the current SmPC: small intestinal perforation (Parker et al., 2013). Notably, this study also uncovered several potential new safety signals (i.e., unexpected AEs) that have not been adequately highlighted in the SmPC or previous clinical studies. These include intestinal ischemia, prolonged activated partial thromboplastin time, increased platelet count, decreased blood chloride, and increased blood urea, among others, which warrant further clinical attention.

### 3.4 Distribution of AE outcomes at the PT level

**FIGURE 4:**
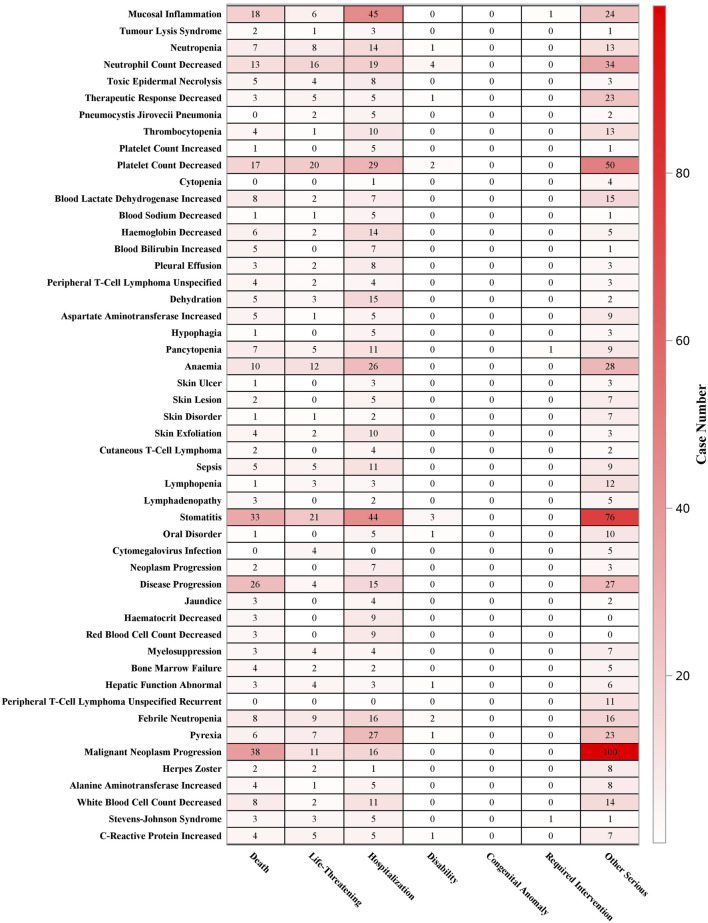
Outcome distribution of the top 50 positive adverse event signals detected by reporting odds ratio, proportional reporting ratio, Bayesian confidence propagation neural network, and multi-item gamma Poisson shrinker algorithms.

Analysis of the outcomes associated with pralatrexate-related AEs revealed differences in the distribution of leading causative events across outcome categories (Figure 4). Specifically, disease progression and stomatitis were the most frequently reported events linked to fatal outcomes. For life-threatening outcomes, stomatitis, decreased platelet count, and decreased neutrophil count were the primary causative events. The top three events leading to hospitalization were mucosal inflammation, stomatitis, and decreased platelet count. Disability outcomes were mainly attributed to decreased neutrophil count, stomatitis, and decreased platelet count, whereas outcomes requiring intervention were exclusively caused by Stevens-Johnson syndrome, pancytopenia, and mucosal inflammation. Other serious outcomes were often associated with malignant neoplasm progression, stomatitis, and decreased platelet count. Overall, the analysis identified stomatitis, decreased platelet count, and decreased neutrophil count as the key risk factors for critical severe outcomes such as death, life-threatening events, and hospitalization.

### 3.5 Time-to-onset analysis of pralatrexate-associated AEs and weibull shape parameter test

**FIGURE 5:**
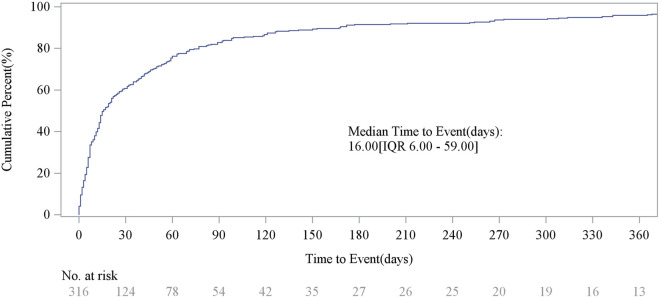
Time to onset of pralatrexate-associated adverse events.

We performed the Weibull shape parameter test for 316 cases of pralatrexate-associated AEs with complete time records (Table 2). The median time to AE onset was 16 days, with an interquartile range of 6–59 days. Both the shape parameter (β) of the Weibull model and the upper limit of its CI were <1, consistent with an early failure pattern. This indicates that the risk of AEs decreases with prolonged treatment duration. As shown in Figure 5, the specific time distribution revealed that the period within 30 days post-administration represented the high-incidence phase for AEs. Between 31 and 180 days, the incidence gradually declined, and the incidence rate showed a rebound from 181 to 360 days. Notably, AEs continued to occur even after more than 1 year of treatment. These temporal characteristics provide critical evidence for pralatrexate safety management and will support clinicians in making timely treatment adjustments to reduce adverse reactions and optimize therapeutic benefits.

**TABLE 2:** Time-to-onset analysis using the Weibull distribution test.

	​		Weibull distribution				Failure type
	TTO (days)		Scale parameter		Shape parameter		
Drug	n	median (IQR)	α	95% CI	β	95% CI	
Pralatrexate	316	16.00 (6.00,59.00)	45.74	37.86–55.27	0.63	0.58–0.69	Early failure

TTO, Time-to-onset; IQR, interquartile range; CI, confidence interval.

### 3.6 Subgroup analysis

#### 3.6.1 Sex subgroup

**FIGURE 6:**
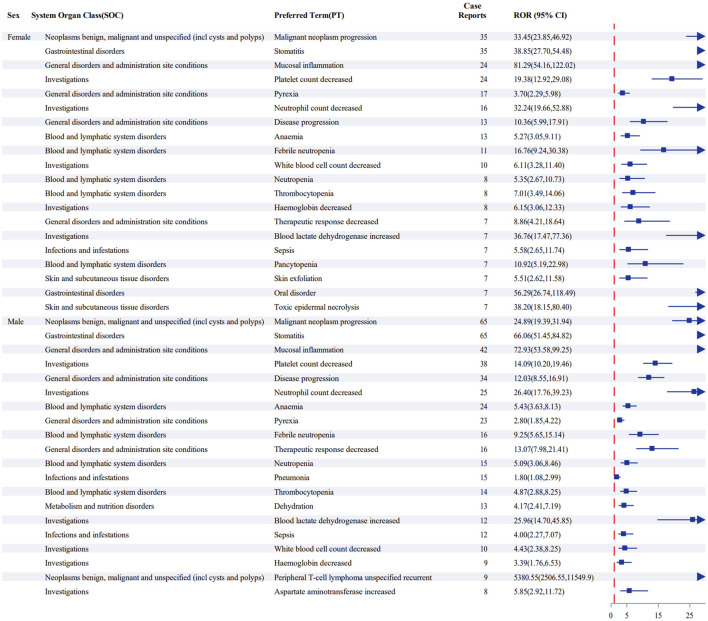
Top 20 preferred terms by frequency in sex-based subgroups after screening for positive signals by reporting odds ratio.

**FIGURE 7:**
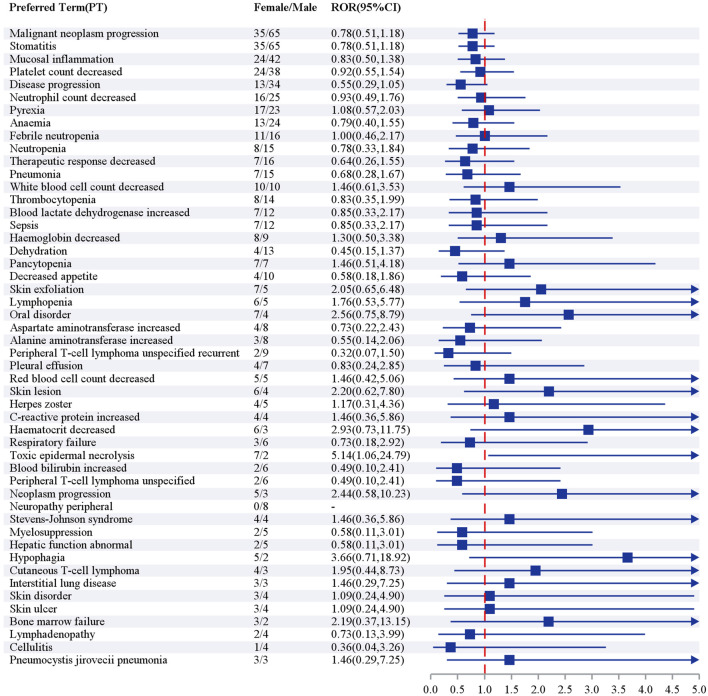
Sex-based risk analysis for preferred terms among the top 50 patients using the reporting odds ratio (95% confidence interval) method.

Positive signal analysis based on the ROR revealed a high degree of similarity between the high-frequency AE signal profiles of male and female patients, with an 80% (16/20) overlap rate for the top 20 PTs (Figure 6). Sex-specific signal detection at the PT level (Figure 7). The analysis revealed that only tumor lysis syndrome showed a significantly higher risk in female recipients (ROR, 5.14; 95% CI, 1.06–24.79). None of the other AEs reached the threshold for significant sex-based differences. This result is consistent with the volcano plot adjusted for false discovery rate (Supplementary Figure S2 in Supplementary Material 3), which demonstrated an absence of widespread sex disparities. Collectively, the results suggest an overall consistent safety profile for pralatrexate across sexes. The only observed exception was for tumor lysis syndrome, which may exhibit a sex-specific risk profile.

#### 3.6.2 Age subgroup

**FIGURE 8:**
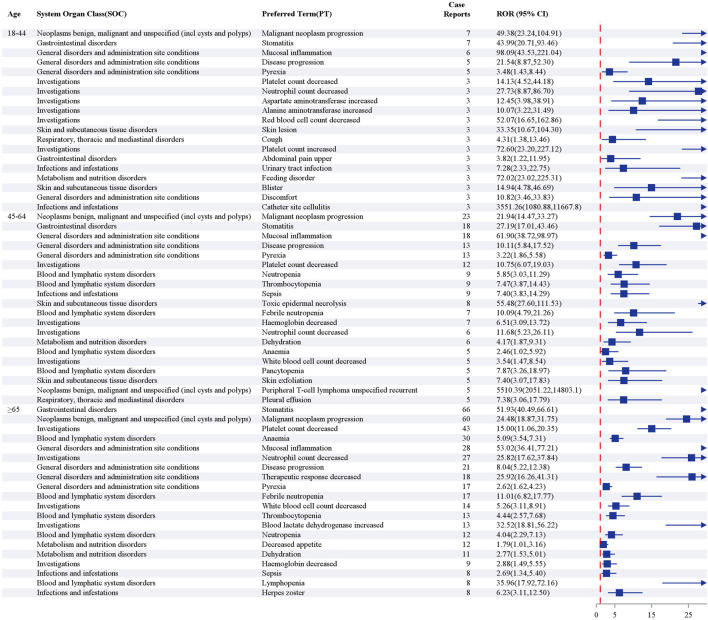
Top 20 preferred terms by frequency in age subgroups after screening for positive signals by reporting odds ratio.

In the age-stratified analysis, differences were observed in the high-frequency AEs of various age groups after screening positive signals using the ROR method (Figure 8). Among the top 20 most frequently reported PTs, stomatitis was the most common event in patients aged ≥65 years, whereas malignant neoplasm progression was the predominant PT in those aged <65 years. Further analysis revealed that a decrease in therapeutic response, increase in blood lactate dehydrogenase, decrease in appetite, lymphocytopenia, and herpes zoster were signals unique to patients aged ≥65 years. In contrast, an increase in aspartate aminotransferase, increase in alanine aminotransferase, skin lesions, cough, an increase in platelet count, upper abdominal pain, urinary tract infection, feeding disorder, blisters, discomfort, and catheter-site cellulitis were exclusively observed in the 18–44 years age group. Additionally, anemia, a decrease in hemoglobin, sepsis, dehydration, and a decrease in white blood cell count tended to cluster in patients aged ≥45 years. These results indicate that the AE profile of pralatrexate exhibits a marked age-dependent pattern: patients aged ≥65 years were more susceptible to mucosal injury and metabolic disturbances, whereas those aged <65 years predominantly experienced tumor progression and liver function abnormalities.

#### 3.6.3 Report type subgroup

**FIGURE 9:**
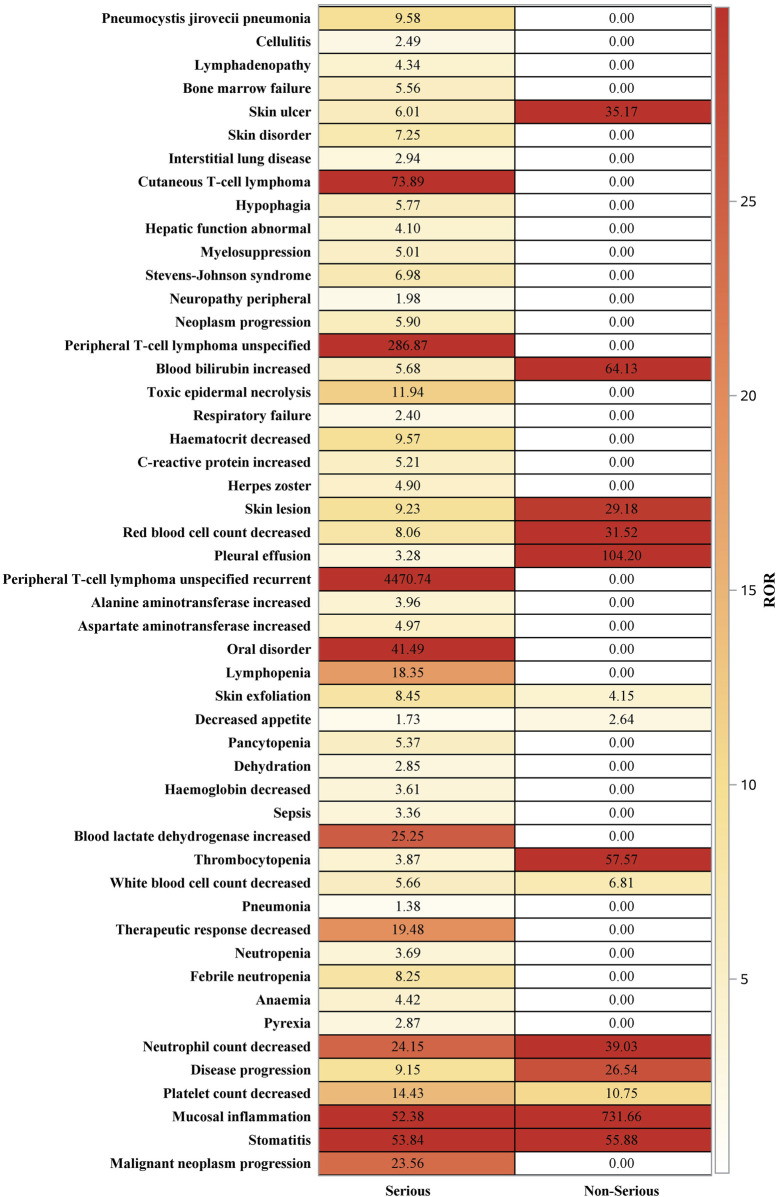
Distribution of signals across report types for the top 50 preferred terms after screening for positive signals by reporting odds ratio.

Results from the report type subgroup analysis (Figure 9) indicated that, in the serious reports group, pralatrexate was associated with a higher frequency of adverse reactions, such as mucositis, oral disorders, elevated blood lactate dehydrogenase, decreased neutrophil count, and lymphocytopenia, compared with other drugs. In the non-serious reports group, pralatrexate was more frequently linked to mucositis, thrombocytopenia, elevated blood bilirubin, decreased neutrophil count, and skin lesions. This analysis revealed the severity-specific risk profile of AEs associated with pralatrexate.

## 4 Discussion

This study represents the first comprehensive and systematic pharmacovigilance analysis of postmarketing AEs associated with pralatrexate based on the FAERS database, revealing the AE profile of pralatrexate in terms of demographic distribution, organ specificity, temporal patterns, and risk stratification. The research not only further validated known risks but also identified multiple potential new safety signals that have not been sufficiently covered by existing data, providing critical evidence for clinical medication safety. The following sections will integrate and discuss the results from four distinct dimensions.

### 4.1 Population and regional characteristics and key risk management priorities

This study identified distinct demographic, geographic, and temporal characteristics in pralatrexate-associated AE reports. We found that the patient population was predominantly male (58.44%) and ≥65 years old (45.65%), a distribution consistent with the epidemiological profile of its indicated condition, relapsed/refractory PTCL, which has a median diagnosis age of 60 years and a higher incidence among men (Swerdlow et al., 2016; Civallero et al., 2024). In contrast, reported adverse reactions to methotrexate, a drug in the same class, are significantly more common in female recipients than in male recipients. This discrepancy is primarily attributed to its widespread use in autoimmune diseases and the distinctive characteristics of female immune responses (Liu et al., 2025). Geographically, the United States (41.56%) and Japan (32.15%) together accounted for 73.71% of all reports. This pattern may stem from two primary factors. First, the United States, where the drug was first developed and marketed, has both a long history of clinical use and a broad patient population. Second, Japan’s revision of the Pharmaceutical Affairs Law has enforced stringent postmarketing monitoring measures, leading to a significant increase in proactive reporting rates (Malik et al., 2010; Ministry of Health, Labour and Welfare of Japan, 2013). Temporal analysis indicated that 34.10% of AEs occurred within 30 days post-administration; together with a high hospitalization rate of 43.16%, this suggests an acute toxicity risk associated with pralatrexate. This toxicity profile is closely linked to its pharmacological mechanism as a high-affinity DHFR inhibitor, which rapidly disrupts DNA synthesis in proliferating cells during the first cell cycle (O'Connor et al., 2007; U.S. Food and Drug Administration, 2020). Therefore, we recommend preventive interventions early in the treatment course to reduce the risk of serious AEs during the initial phase.

### 4.2 Analysis of SOCs indicated multi-system toxicity

This study, through SOC analysis, revealed the multi-system distribution of pralatrexate-associated AEs. The involvement of 25 SOC categories confirms the drug’s broad biological effects. The high incidence of systemic and administration site reactions (15.80%) is partly related to the drug’s non-selective inhibition of DHFR. This mechanism disrupts DNA synthesis in tumor cells and also affects rapidly proliferating normal cells, such as mucosal and epidermal basal cells, leading to characteristic systemic reactions (Malik et al., 2010; U.S. Food and Drug Administration, 2020). Another contributing factor is local adverse reactions directly related to intravenous injection procedures (Kumar et al., 2001). A key finding was that hematological and hepatic toxicities were the most common types of laboratory abnormalities (15.35%). This highlights the importance of vigilant monitoring of blood counts and liver function in clinical practice. Compared with methotrexate, pralatrexate exhibits distinct hepatorenal toxicity profiles. Renal toxicities associated with methotrexate, such as hematuria and proteinuria, are dose- and duration-dependent, with long-term methotrexate use potentially leading to chronic kidney injury and hepatic fibrosis (Liu et al., 2025; Visser and van der Heijde, 2009). In contrast, pralatrexate is associated with fewer reports of renal toxicity, and its primary associated hepatic injury is elevated transaminase levels, with no clear evidence of liver fibrosis risk. This divergence likely stems from differences in the tissue distribution of methotrexate and pralatrexate or their respective metabolic pathways (U.S. Food and Drug Administration, 2024).

The incidence rates of hematologic and lymphatic system disorders (7.94%) and infectious and infestation diseases (8.39%) indicate that chemotherapy increases immunodeficiency and infection risk by causing both neutropenia and mucosal barrier damage (O'Connor et al., 2009; Mould et al., 2009). These findings suggest that systematic implementation of prophylactic anti-infection strategies is necessary during clinical treatment. While both pralatrexate and methotrexate induce myelosuppression through DNA synthesis inhibition, their hematologic toxicity profiles differ. Pralatrexate hematotoxicity presents with acute onset, whereas methotrexate hematologic AEs often feature progressive damage associated with dose and duration dependency (U.S. Food and Drug Administration, 2024). Notably, in this study, pralatrexate did not produce musculoskeletal toxicities similar to those of methotrexate, such as arthralgia or osteoporosis (Liu et al., 2025), suggesting that pralatrexate may have a lesser impact on fibroblasts or bone metabolism.

Tumor-related events (9.10%) primarily manifested as recurrence or progression of the primary disease. However, available data cannot definitively distinguish whether these are attributable to the natural history of the disease, potential drug effects, or other confounding factors. Although the highly aggressive nature of PTCL may partly explain the high incidence of such events, several possibilities require careful consideration. For example, differences in baseline tumor burden and concomitant treatments may affect event attribution; drug-induced modulation of the immune microenvironment may indirectly contribute to disease progression; and tumor progression may itself indicate insufficient drug efficacy. It should be noted that this study is limited by the inherent biases of retrospective analysis and lacks pathological or molecular evidence to establish causality. Future studies incorporating dynamic efficacy assessments and time-series analyses of AEs could improve the reliability of the attribution judgments.

### 4.3 The identification of positive signals suggests unanticipated clinical risks

This study utilized a multi-algorithm signal detection approach to reveal the complex safety profile of pralatrexate in real-world settings. It not only confirmed the known adverse reactions previously documented in the drug labels and clinical trials, but also identified several potential new safety signals that have not yet been fully covered by the existing data.

Myelosuppression, mucositis, and dermatologic reactions are well-documented toxicities of pralatrexate, with their mechanisms closely linked to its antimetabolite properties. As a DHFR inhibitor, pralatrexate competitively depletes intracellular reduced folate, thereby disrupting thymidine and purine synthesis and inhibiting DNA replication. Given the high proliferative rates of hematopoietic cells, gastrointestinal mucosal cells, and skin basal cells, these tissues exhibit heightened sensitivity to DNA synthesis inhibitors, predisposing patients to hematologic, gastrointestinal, and dermatologic AEs, a pattern consistent with previous clinical observations (Parker et al., 2013; O'Connor et al., 2011; Hong et al., 2019). Furthermore, intracellular accumulation of pralatrexate polyglutamates exacerbates toxicity to mucosal basal cells, impairing epithelial repair capacity (Toner et al., 2006; Marchi et al., 2010). Early Phase I studies correlated elevated homocysteine and methylmalonic acid levels with mucositis development, while subsequent trials demonstrated that folic acid and vitamin B12 supplementation effectively reduce both mucositis incidence and severity (O’Connor et al., 2009; Mould et al., 2009). Although this supplementation strategy resembles leucovorin rescue after methotrexate, the specific protocols differ. Methotrexate-induced mucosal toxicity is dose- and duration-dependent, and rescue is achieved by post-methotrexate leucovorin administration (U.S. Food and Drug Administration, 2024). In contrast, owing to pralatrexate’s rapid inhibition of DHFR, folic acid intervention must be initiated 10 days before the first pralatrexate dose (U.S. Food and Drug Administration, 2020).

Tumor lysis syndrome is an adverse reaction explicitly listed on the pralatrexate drug labels. Its mechanism primarily stems from the drug’s potent and rapid cytotoxic effect on high-burden, highly proliferative PTCL tumor cells. This leads to the massive release of intracellular contents into the bloodstream, overwhelming the body’s metabolic capacity and consequently triggering life-threatening metabolic disturbances (Gupta and Moore, 2018). Therefore, for patients with high-risk factors, a risk assessment should be conducted prior to treatment, accompanied by preventive strategies including hydration, urate-lowering therapy, and close monitoring of complete blood count, hepatic and renal function, cardiac enzymes, and electrolytes (Howard et al., 2024). Furthermore, the drug labels also clearly highlight the risk of liver function abnormalities, the primary clinical manifestation of which is elevated serum transaminases. Although the exact mechanism remains unclear, pharmacokinetic studies indicate that approximately 34% of the administered dose is renally excreted unchanged, suggesting that a portion of the drug may still undergo hepatic metabolism. This process could induce hepatocyte injury or functional disturbances, leading to the abnormal release of liver enzymes. In accordance with the pralatrexate prescribing information, regular monitoring of liver function is mandated during treatment. Furthermore, the label specifies that dose adjustment or temporary discontinuation should be promptly implemented for persistent abnormalities, with the specific strategy determined by the severity grade (U.S. Food and Drug Administration, 2020).

The disproportionality signals for intestinal ischemia and small intestinal perforation may be mechanistically linked to pralatrexate-induced direct damage to the intestinal mucosal barrier. The PROPEL trial documented a fatal case of bowel perforation secondary to sepsis (O'Connor et al., 2009), yet this risk has not been included in the current drug labels. Based on our finding of a high incidence of gastrointestinal AEs, we recommend implementing dynamic monitoring of gastrointestinal function during treatment with pralatrexate. The signals for decreased blood chloride levels, hypoalbuminemia, and malnutrition are likely associated with reduced food intake caused by the drug. The newly identified signals for prolonged activated partial thromboplastin time and increased platelet count suggest potential coagulation abnormalities associated with this drug. As such, it is advisable to enhance coagulation function monitoring during treatment.

This study detected multiple high-intensity signals, including those for relapsed PTCL, tumor-associated fever, disease progression, and catheter-site cellulitis. Relapsed PTCL, tumor-associated fever, and disease progression are likely to be associated with the underlying primary disease (Eyre et al., 2025), suggesting that clinicians should carefully differentiate between drug effects and natural disease progression. The occurrence of catheter-site cellulitis may involve both drug-induced immunosuppression and catheter manipulation practices, highlighting the need to strengthen the prevention and control of device-related infections during periods of significant myelosuppression.

Furthermore, analysis of AE outcomes revealed that mucositis and hematologic toxicity were the primary factors leading to severe outcomes such as death, life-threatening conditions, and hospitalization. Subgroup analysis based on report types further supported this conclusion. These findings suggest that, in clinical practice, emphasis should be placed on preventing the risks of myelosuppression and mucosal injury. Additionally, standardized supplementation of vitamin B12 and folic acid should be implemented during the early stages of medication, while enhanced monitoring of adverse reactions should be maintained throughout the entire treatment course. Timely dosage modification will also be crucial for reducing the incidence and severity of related AEs.

### 4.4 Time-series and population-based risk management

This study, through time-series analysis and stratified analyses by sex and age, revealed the temporal dynamics and population heterogeneity of pralatrexate-associated AEs, providing evidence-based support for the development of differentiated risk management strategies in clinical practice.

#### 4.4.1 Dynamic monitoring based on temporal patterns

The temporal characteristics of pralatrexate-associated AEs indicate a high-risk period within 30 days post-administration, with a median time of 16 days. Weibull analysis suggested that the risk decreases with prolonged treatment duration, highlighting the need for intensified monitoring strategies early in therapy. However, a rebound in risk was observed after 180 days of medication. We recommend that patients receiving pralatrexate undergo extended follow-up for at least 12 months beyond the end of treatment. Regular medical evaluations during this period can help mitigate the impact of adverse reactions and improve the risk–benefit ratio.

#### 4.4.2 Risk management and prevention in sex-based subgroups

Although this study indicates that the overall safety profile of pralatrexate is generally consistent between male and female patients, female patients have a higher risk of toxic epidermal necrolysis. The pathogenesis of toxic epidermal necrolysis suggests that this phenomenon may be partially attributable to sex-based differences in the pharmacokinetics of pralatrexate (Mockenhaupt et al., 2008). While previous studies have not directly confirmed such variations, the existing literature points to limitations in the current body surface area-based dosing strategy for antitumor drugs. Owing to common physiological disparities in body composition and key pharmacokinetic parameters between sexes, body surface area-based dosing may lead to higher drug plasma concentrations in female patients, potentially resulting in more severe drug toxicities (Ozdemir, 2024). This discrepancy highlights the need for sex-specific risk prevention and management strategies in clinical practice. For female patients receiving pralatrexate, we recommend enhanced early monitoring of skin symptoms, such as mucosal erythema or epidermal detachment. Early recognition and prompt intervention should be implemented upon the emergence of precursor symptoms, with dose adjustment considered when necessary.

#### 4.4.3 Age-stratified risk management and prevention

This study confirmed that pralatrexate-associated AEs exhibit significant age-related heterogeneity, which may be related to dynamic interactions between age-related physiological and pathological characteristics. In patients aged ≥65 years, stomatitis was the most frequently reported AE. This group also showed a lower therapeutic response, higher blood lactate dehydrogenase level, and increased risk of herpes zoster infection. These findings may be associated with an age-related decline in mucosal repair capacity, increased tumor burden, and weakened immune function (Zhang et al., 2024; Kroemer et al., 2025; Claps et al., 2022; Chen et al., 2024). Notably, the toxicity profile of pralatrexate differs from that of methotrexate in patients ≥65 years old, with recipients of the latter more likely to experience drug allergies and immune-related AEs (Liu et al., 2025). In contrast, patients aged 18–44 were more prone to abnormal liver function and skin toxicities, suggesting that the drug may directly damage highly metabolically active hepatocytes and rapidly proliferating skin and mucosal cells (Parker et al., 2013). Among patients aged ≥45, there were higher incidences of hematologic toxicity and severe infectious complications, likely attributable to declining bone marrow hematopoietic function and progressive immune deterioration leading to reduced tolerance to chemotherapy (Dolan et al., 2025). Based on these findings, we recommend establishing an age-stratified toxicity monitoring system in clinical practice, focusing on mucosal and immune function in older patients, liver function in younger adults, and hematologic parameters in patients ≥45 years old, to optimize individualized risk management. The age subgroup analysis in this study adopted conventional stratification, which may not fully capture risk variations within specific age ranges of patients with PTCL. Future studies should consider a more refined stratification strategy based on the age distribution characteristics of PTCL.

This study conducted a pharmacovigilance analysis for pralatrexate using the FAERS spontaneous reporting system. It is important to acknowledge the inherent limitations of FAERS as a passive surveillance platform. First, underreporting is prevalent, particularly in non-English-speaking regions, and overreporting risks persist owing to confounding factors such as concomitant medications (Yan et al., 2025). Second, the FAERS database lacks comprehensive clinical records, omitting critical medical information including detailed patient histories and disease severity, with incomplete documentation of drug dosage, administration routes, and treatment duration (Zhou et al., 2023). Third, safety signals identified through data mining only indicate statistical associations between drugs and AEs, neither quantifying real-world risk occurrence nor establishing causal relationships (Shi et al., 2025). Therefore, the identified potential risk signals require validation through high-quality prospective multicenter clinical studies.

This study identified 2,241 reports of AEs with pralatrexate as the PS drug from the FAERS database, uncovering 84 safety signals through a four-fold disproportionality analysis algorithm. These signals span 25 SOCs. Through in-depth multidimensional analysis, which considered the characteristics of system organ distribution, PT level, temporal distribution, and subgroup differences by sex, age, and report type, this research systematically constructed the first comprehensive safety profile of pralatrexate. The findings provide critical evidence-based support for optimizing clinical risk monitoring systems and informing prevention and control strategies.

## Data Availability

The original contributions presented in the study are included in the article/Supplementary Material, further inquiries can be directed to the corresponding author.
