# Supplementary material for: Real-world pharmacovigilance analysis of pralatrexate using the FDA adverse event reporting system database
Source: Front Pharmacol. 2026 Apr 8;17:1773445. doi: 10.3389/fphar.2026.1773445 (PMC13099823; doi:10.3389/fphar.2026.1773445)
Supplement: Supplementary file 1 [file Supplementaryfile3.docx]

Supplementary Table 1. Example of the deduplicated report

| **primaryid** | **caseid** | **FDA_DT** | **operation** |
| --- | --- | --- | --- |
| 4271953 | 4070800 | 20040113 | removal |
| 4271960 | 4070800 | 20040113 | removal |
| 4283861 | 4070800 | 20040130 | removal |
| 4314767 | 4070800 | 20040308 | retention |

Supplementary Table 2. Four-fold table of disproportionality analyses

|  | Pralatrexate-related AEs | Non-pralatrexate-related AEs | Total |
| --- | --- | --- | --- |
| Pralatrexate | a | b | a+b |
| Non-pralatrexate | c | d | c+d |
| Total | a+c | b+d | N=a+b+c+d |

AEs, adverse events; a, Number of target AEs caused by pralatrexate; b, Number of non-target AEs caused by pralatrexate; c, Number of target AEs caused by drugs other than pralatrexate; d, Number of non-target AEs caused by drugs other than pralatrexate; N, Total number of AEs included in background database for analysis.

Supplementary Table 3. Four calculation formulas and positive thresholds for detecting pralatrexate AE signals

| **Algorithms** | **Equation** | **Criteria** |
| --- | --- | --- |
| ROR | 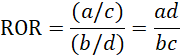  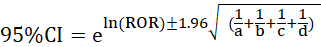 | a≥3 and lower limit of 95% CI > 1 |
| PRR | 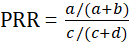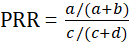  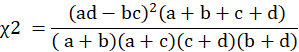 | a≥3, PRR≥2, and χ²≥4 |
| BCPNN | IC=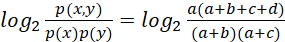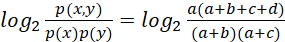  E(IC)=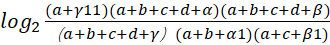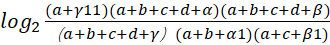  V(IC)=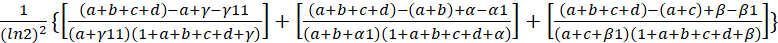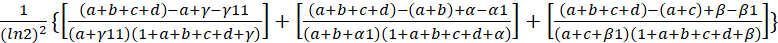  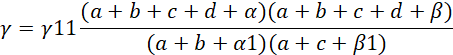  *IC-2SD=E(IC)-2*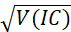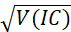  α1=β1=1;α=β=2;γ11=1 | IC-2SD>0 |
| MGPS | 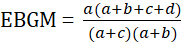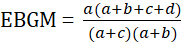  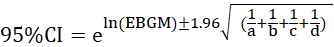 | EBGM05>2 |

CI, confidence interval; IC, information component; IC-2SD, lower limit of the 95% CI of the IC; EBGM05, lower limit of the empirical Bayes geometric mean 95% CI.


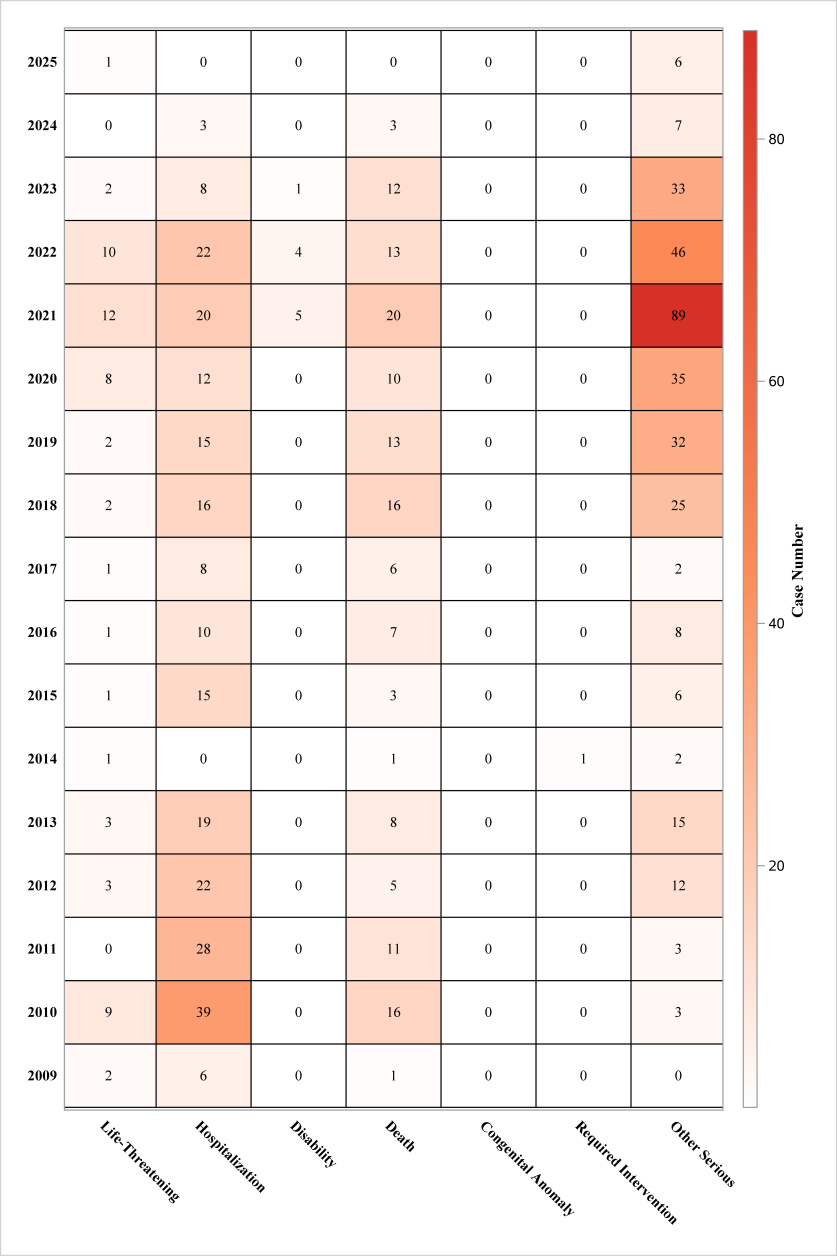


Supplementary Figure 1 . Annual stratified data on outcomes associated with pralatrexate-related adverse events.

Supplementary Table 4. Signal strength and expectedness classification of adverse events at the preferred term level ranked by reporting odds ratio

| **SOC** | **PT** | **Case reports** | **ROR (95% CI)** | **PRR (χ2)** | **IC**  **(IC-2SD)** | **EBGM (EBGM05)** | **Expectedness classification*** |
| --- | --- | --- | --- | --- | --- | --- | --- |
| Neoplasms benign, malignant and unspecified (incl cysts and polyps) | Peripheral T-cell lymphoma unspecified recurrent | 11 | 6173.47  (3189.01,11951.0) | 6143.17  (54283.6) | 12.27  (2.66) | 4936.67  (2550.12) | E |
| Neoplasms benign, malignant and unspecified (incl cysts and polyps) | Peripheral T-cell lymphoma unspecified | 8 | 397.96  (197.69,801.11) | 396.55  (3107.39) | 8.61  (2.17) | 390.40  (193.94) | E |
| Infections and infestations | Catheter site cellulitis | 3 | 341.60  (109.25,1068.13) | 341.15  (1003.83) | 8.39  (0.53) | 336.59  (107.65) | E |
| Neoplasms benign, malignant and unspecified (incl cysts and polyps) | Tumour associated fever | 4 | 280.51  (104.62,752.15) | 280.01  (1099.82) | 8.11  (1.00) | 276.94  (103.28) | E |
| Neoplasms benign, malignant and unspecified (incl cysts and polyps) | T-cell lymphoma | 4 | 103.18  (38.61,275.71) | 103.00  (402.38) | 6.68  (0.97) | 102.58  (38.39) | E |
| Neoplasms benign, malignant and unspecified (incl cysts and polyps) | Cutaneous T-cell lymphoma | 7 | 102.71  (48.84,216.03) | 102.40  (700.02) | 6.67  (1.88) | 101.99  (48.49) | E |
| General disorders and administration site conditions | Mucosal inflammation | 68 | 75.02  (58.92,95.54) | 72.78  (4802.00) | 6.18  (4.80) | 72.57  (56.99) | E |
| Gastrointestinal disorders | Stomatitis | 107 | 51.93  (42.75,63.07) | 49.50  (5079.08) | 5.63  (4.81) | 49.40  (40.67) | E |
| Neoplasms benign, malignant and unspecified (incl cysts and polyps) | Epstein-Barr virus associated lymphoproliferative disorder | 3 | 47.33  (15.24,147.02) | 47.27  (135.62) | 5.56  (0.47) | 47.18  (15.19) | E |
| General disorders and administration site conditions | Mucosal disorder | 4 | 45.50  (17.05,121.46) | 45.42  (173.48) | 5.50  (0.91) | 45.34  (16.99) | E |
| Investigations | Staphylococcus test positive | 4 | 45.10  (16.90,120.37) | 45.02  (171.86) | 5.49  (0.91) | 44.94  (16.84) | E |
| Gastrointestinal disorders | Small intestinal perforation | 3 | 44.99  (14.48,139.74) | 44.93  (128.63) | 5.49  (0.46) | 44.85  (14.44) | E |
| Gastrointestinal disorders | Oral disorder | 12 | 42.26  (23.95,74.56) | 42.04  (479.99) | 5.39  (2.53) | 41.97  (23.79) | E |
| Investigations | Blood lactate dehydrogenase increased | 20 | 33.43  (21.52,51.94) | 33.14  (622.82) | 5.05  (3.08) | 33.10  (21.31) | E |
| Neoplasms benign, malignant and unspecified (incl cysts and polyps) | Malignant neoplasm progression | 110 | 32.44  (26.78,39.29) | 30.89  (3183.02) | 4.95  (4.32) | 30.86  (25.47) | E |
| Investigations | Blood chloride decreased | 3 | 30.68  (9.88,95.25) | 30.64  (85.90) | 4.94  (0.42) | 30.60  (9.85) | U |
| Investigations | Neutrophil count decreased | 42 | 29.89  (22.02,40.57) | 29.35  (1149.55) | 4.87  (3.70) | 29.32  (21.60) | E |
| Injury, poisoning and procedural complications | Wound secretion | 4 | 27.57  (10.33,73.55) | 27.52  (102.11) | 4.78  (0.83) | 27.49  (10.30) | E |
| Blood and lymphatic system disorders | Lymphopenia | 12 | 23.94  (13.57,42.23) | 23.82  (262.14) | 4.57  (2.31) | 23.80  (13.49) | E |
| Infections and infestations | Oesophageal candidiasis | 3 | 21.35  (6.88,66.30) | 21.33  (58.08) | 4.41  (0.37) | 21.31  (6.86) | E |
| Metabolism and nutrition disorders | Tumour lysis syndrome | 6 | 19.71  (8.84,43.93) | 19.66  (106.19) | 4.30  (1.33) | 19.64  (8.81) | E |
| Skin and subcutaneous tissue disorders | Toxic epidermal necrolysis | 9 | 16.81  (8.73,32.36) | 16.75  (133.21) | 4.07  (1.79) | 16.74  (8.70) | E |
| Investigations | Platelet count decreased | 63 | 16.69  (12.99,21.45) | 16.25  (902.80) | 4.02  (3.35) | 16.24  (12.64) | E |
| Investigations | Activated partial thromboplastin time prolonged | 3 | 15.65  (5.04,48.57) | 15.63  (41.06) | 3.97  (0.30) | 15.62  (5.03) | U |
| Skin and subcutaneous tissue disorders | Dermatitis bullous | 4 | 15.37  (5.76,41.00) | 15.34  (53.60) | 3.94  (0.70) | 15.33  (5.75) | E |
| Gastrointestinal disorders | Intestinal ischaemia | 3 | 15.20  (4.90,47.17) | 15.18  (39.71) | 3.92  (0.29) | 15.17  (4.89) | U |
| Injury, poisoning and procedural complications | Skin abrasion | 4 | 15.09  (5.66,40.25) | 15.06  (52.49) | 3.91  (0.69) | 15.05  (5.64) | U |
| Gastrointestinal disorders | Cheilitis | 3 | 15.00  (4.83,46.57) | 14.99  (39.14) | 3.90  (0.29) | 14.98  (4.83) | E |
| Metabolism and nutrition disorders | Hypoalbuminaemia | 4 | 14.80  (5.55,39.47) | 14.77  (51.34) | 3.88  (0.68) | 14.76  (5.53) | E |
| Investigations | Blood uric acid increased | 3 | 14.47  (4.66,44.90) | 14.45  (37.54) | 3.85  (0.28) | 14.44  (4.65) | E |
| Infections and infestations | Pneumocystis jirovecii pneumonia | 6 | 13.46  (6.04,30.00) | 13.43  (68.99) | 3.75  (1.18) | 13.42  (6.02) | E |
| Blood and lymphatic system disorders | Cytopenia | 5 | 12.87  (5.35,30.96) | 12.85  (54.60) | 3.68  (0.93) | 12.84  (5.34) | E |
| Investigations | Platelet count increased | 6 | 12.50  (5.61,27.86) | 12.47  (63.28) | 3.64  (1.15) | 12.46  (5.59) | U |
| Investigations | Haematocrit decreased | 9 | 12.03  (6.25,23.15) | 11.98  (90.59) | 3.58  (1.60) | 11.98  (6.22) | E |
| Blood and lymphatic system disorders | Febrile neutropenia | 27 | 11.59  (7.93,16.94) | 11.46  (258.02) | 3.52  (2.51) | 11.46  (7.84) | E |
| General disorders and administration site conditions | Disease progression | 48 | 11.54  (8.67,15.36) | 11.31  (451.84) | 3.50  (2.81) | 11.31  (8.49) | E |
| General disorders and administration site conditions | Therapeutic response decreased | 23 | 10.82  (7.18,16.33) | 10.72  (202.90) | 3.42  (2.34) | 10.72  (7.11) | E |
| Skin and subcutaneous tissue disorders | Skin lesion | 10 | 10.36  (5.57,19.28) | 10.32  (84.15) | 3.37  (1.61) | 10.31  (5.54) | E |
| Investigations | Blood albumin decreased | 3 | 10.30  (3.32,31.98) | 10.29  (25.15) | 3.36  (0.19) | 10.29  (3.31) | E |
| Infections and infestations | Skin infection | 4 | 10.11  (3.79,26.96) | 10.09  (32.76) | 3.33  (0.55) | 10.09  (3.78) | E |
| Skin and subcutaneous tissue disorders | Skin reaction | 5 | 10.01  (4.16,24.08) | 9.99  (40.44) | 3.32  (0.82) | 9.99  (4.15) | E |
| Skin and subcutaneous tissue disorders | Stevens-Johnson syndrome | 8 | 9.78  (4.89,19.59) | 9.75  (62.82) | 3.29  (1.34) | 9.75  (4.87) | E |
| Investigations | Red blood cell count decreased | 10 | 9.57  (5.14,17.81) | 9.53  (76.34) | 3.25  (1.55) | 9.52  (5.12) | E |
| Respiratory, thoracic and mediastinal disorders | Lung infiltration | 3 | 8.81  (2.84,27.34) | 8.80  (20.73) | 3.14  (0.13) | 8.79  (2.83) | E |
| Infections and infestations | Oral candidiasis | 4 | 8.78  (3.29,23.43) | 8.77  (27.54) | 3.13  (0.49) | 8.77  (3.29) | E |
| Immune system disorders | Haemophagocytic lymphohistiocytosis | 3 | 8.74  (2.82,27.13) | 8.73  (20.53) | 3.13  (0.13) | 8.73  (2.81) | E |
| Infections and infestations | Cytomegalovirus infection | 5 | 8.36  (3.47,20.10) | 8.34  (32.29) | 3.06  (0.73) | 8.34  (3.47) | E |
| Respiratory, thoracic and mediastinal disorders | Atelectasis | 3 | 8.28  (2.67,25.69) | 8.27  (19.16) | 3.05  (0.11) | 8.26  (2.66) | U |
| Investigations | Blood bilirubin increased | 8 | 8.02  (4.01,16.06) | 8.00  (48.98) | 3.00  (1.21) | 7.99  (3.99) | E |
| Blood and lymphatic system disorders | Bone marrow failure | 6 | 7.81  (3.50,17.39) | 7.79  (35.49) | 2.96  (0.89) | 7.78  (3.49) | E |
| General disorders and administration site conditions | Mass | 4 | 7.79  (2.92,20.77) | 7.78  (23.62) | 2.96  (0.43) | 7.77  (2.91) | E |
| Metabolism and nutrition disorders | Malnutrition | 3 | 7.58  (2.44,23.51) | 7.57  (17.09) | 2.92  (0.07) | 7.56  (2.44) | E |
| Metabolism and nutrition disorders | Feeding disorder | 5 | 7.55  (3.14,18.16) | 7.53  (28.34) | 2.91  (0.67) | 7.53  (3.13) | E |
| Blood and lymphatic system disorders | Pancytopenia | 15 | 7.52  (4.53,12.50) | 7.48  (84.21) | 2.90  (1.69) | 7.48  (4.50) | E |
| Skin and subcutaneous tissue disorders | Skin ulcer | 7 | 7.48  (3.56,15.72) | 7.46  (39.19) | 2.90  (1.02) | 7.46  (3.55) | E |
| Investigations | Blood sodium decreased | 5 | 7.29  (3.03,17.53) | 7.27  (27.05) | 2.86  (0.65) | 7.27  (3.02) | U |
| Infections and infestations | Bacteraemia | 3 | 7.24  (2.33,22.48) | 7.23  (16.12) | 2.85  (0.05) | 7.23  (2.33) | E |
| Neoplasms benign, malignant and unspecified (incl cysts and polyps) | Lymphoma | 4 | 7.07  (2.65,18.85) | 7.06  (20.79) | 2.82  (0.38) | 7.05  (2.64) | E |
| Blood and lymphatic system disorders | Myelosuppression | 7 | 7.04  (3.35,14.79) | 7.02  (36.16) | 2.81  (0.98) | 7.02  (3.34) | E |
| Investigations | C-reactive protein increased | 9 | 7.03  (3.65,13.53) | 7.01  (46.37) | 2.81  (1.22) | 7.01  (3.64) | E |
| Metabolism and nutrition disorders | Hypophagia | 7 | 6.86  (3.27,14.41) | 6.84  (34.94) | 2.77  (0.96) | 6.84  (3.26) | E |
| Neoplasms benign, malignant and unspecified (incl cysts and polyps) | Metastases to central nervous system | 3 | 6.70  (2.16,20.79) | 6.69  (14.52) | 2.74  (0.02) | 6.69  (2.16) | E |
| Infections and infestations | Device related infection | 4 | 6.55  (2.46,17.48) | 6.54  (18.79) | 2.71  (0.34) | 6.54  (2.45) | E |
| Investigations | Blood urea increased | 4 | 6.29  (2.36,16.77) | 6.28  (17.75) | 2.65  (0.32) | 6.28  (2.35) | U |
| Investigations | Aspartate aminotransferase increased | 12 | 6.18  (3.50,10.90) | 6.15  (51.80) | 2.62  (1.34) | 6.15  (3.49) | E |
| Investigations | Lymphocyte count decreased | 4 | 6.17  (2.32,16.47) | 6.17  (17.31) | 2.62  (0.31) | 6.16  (2.31) | E |
| Neoplasms benign, malignant and unspecified (incl cysts and polyps) | Neoplasm progression | 8 | 5.97  (2.98,11.94) | 5.95  (32.94) | 2.57  (0.98) | 5.95  (2.97) | E |
| Skin and subcutaneous tissue disorders | Skin disorder | 7 | 5.89  (2.80,12.37) | 5.87  (28.31) | 2.55  (0.85) | 5.87  (2.80) | E |
| Investigations | White blood cell count decreased | 23 | 5.86  (3.89,8.84) | 5.81  (91.79) | 2.54  (1.68) | 5.81  (3.85) | E |
| Blood and lymphatic system disorders | Anaemia | 39 | 5.65  (4.12,7.75) | 5.57  (146.60) | 2.48  (1.86) | 5.57  (4.06) | E |
| Blood and lymphatic system disorders | Thrombocytopenia | 22 | 5.53  (3.63,8.42) | 5.49  (80.86) | 2.46  (1.59) | 5.49  (3.61) | E |
| Infections and infestations | Candida infection | 4 | 5.43  (2.03,14.47) | 5.42  (14.41) | 2.44  (0.23) | 5.42  (2.03) | E |
| Hepatobiliary disorders | Hepatic function abnormal | 7 | 5.35  (2.55,11.24) | 5.34  (24.67) | 2.42  (0.77) | 5.34  (2.54) | E |
| Blood and lymphatic system disorders | Neutropenia | 24 | 5.00  (3.35,7.48) | 4.96  (76.05) | 2.31  (1.52) | 4.96  (3.32) | E |
| Hepatobiliary disorders | Jaundice | 5 | 4.98  (2.07,11.99) | 4.98  (15.89) | 2.31  (0.40) | 4.97  (2.07) | E |
| Respiratory, thoracic and mediastinal disorders | Pleural effusion | 11 | 4.92  (2.72,8.90) | 4.90  (34.18) | 2.29  (1.05) | 4.90  (2.71) | E |
| Investigations | Alanine aminotransferase increased | 11 | 4.90  (2.71,8.87) | 4.88  (34.00) | 2.29  (1.05) | 4.88  (2.70) | E |
| Infections and infestations | Sepsis | 19 | 4.71  (3.00,7.41) | 4.68  (55.12) | 2.23  (1.33) | 4.68  (2.98) | E |
| Blood and lymphatic system disorders | Lymphadenopathy | 6 | 4.67  (2.10,10.42) | 4.67  (17.28) | 2.22  (0.52) | 4.66  (2.09) | E |
| Investigations | Haemoglobin decreased | 17 | 4.50  (2.80,7.26) | 4.48  (45.99) | 2.16  (1.22) | 4.48  (2.78) | E |
| Skin and subcutaneous tissue disorders | Skin exfoliation | 13 | 4.43  (2.57,7.63) | 4.41  (34.26) | 2.14  (1.05) | 4.41  (2.55) | E |
| Infections and infestations | Herpes zoster | 9 | 4.30  (2.24,8.28) | 4.29  (22.73) | 2.10  (0.78) | 4.29  (2.23) | E |
| Metabolism and nutrition disorders | Dehydration | 17 | 3.50  (2.17,5.63) | 3.48  (30.05) | 1.80  (0.93) | 3.48  (2.16) | E |
| General disorders and administration site conditions | Pyrexia | 40 | 3.22  (2.35,4.40) | 3.18  (59.97) | 1.67  (1.14) | 3.18  (2.32) | E |

SOC, system organ class; PT, preferred term; ROR, reporting odds ratio; CI, confidence interval; PRR, proportional reporting ratio; IC, information component; IC-2SD, lower limit of the 95% CI of the IC; MGPS, multi-item gamma Poisson shrinker; EBGM05, lower limit of the empirical Bayes geometric mean 95% CI；*Expectedness classification codes: E, Expected adverse events(documented in core authorized documents (such as the Summary of Product Characteristics, SmPCs)or in published literature, explainable by the patients' primary disease, or those that have a clear and direct pathophysiological link to events already documented in the SmPCs); U, Unexpected adverse events


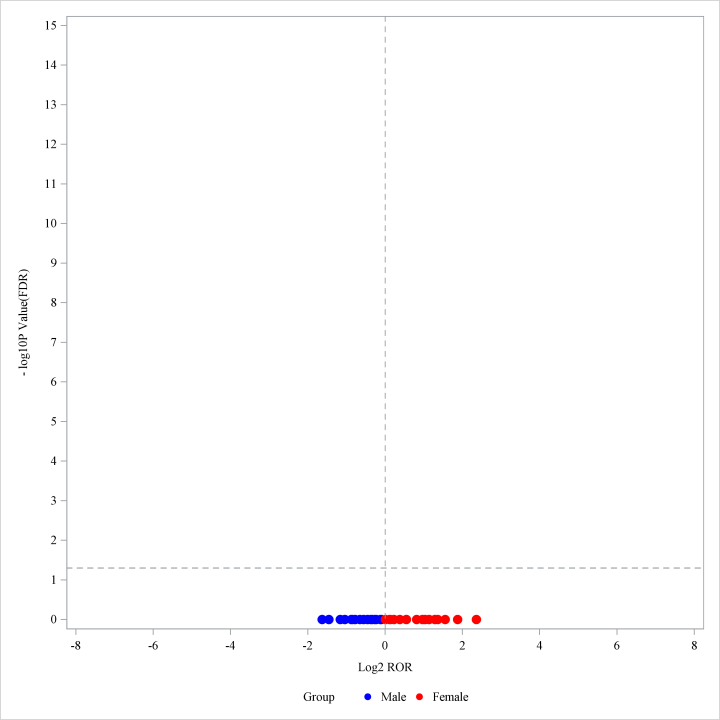


Supplementary Figure 2. Sex difference risk signal volcano map（the p-value is adjusted with false discovery rate(FDR) method）. Red points indicate potential adverse events in female patients, while blue points denote those in male patients.

Supplementary Table 5. List of abbreviations

| **Abbreviation** | **Explanation** |
| --- | --- |
| AE | adverse event |
| BCPNN | Bayesian confidence propagation neural network |
| CASEID | case identifier |
| CI | confidence interval |
| DEMO | demographic and administrative information |
| DHFR | dihydrofolate reductase |
| DRUG | drug information |
| EBGM05 | lower limit of the empirical Bayes geometric mean 95% confidence interval |
| FAERS | FDA Adverse Event Reporting System |
| FDA | Food and Drug Administration |
| FDA_DT | FDA receipt date |
| IC-2SD | lower limit of the 95% confidence interval of the information component |
| INDI | medication administration indications |
| IQR | interquartile range |
| MedDRA | Medical Dictionary for Regulatory Activities |
| MGPS | multi-item gamma Poisson shrinker |
| OUTC | patient outcome information |
| PRIMARYID | primary identifiers |
| PRR | proportional reporting ratio |
| PS | primary suspected |
| PT | preferred term |
| PTCL | peripheral T cell lymphoma |
| REAC | adverse drug reaction information |
| ROR | reporting odds ratio |
| RPSR | reporting source information |
| SmPC | summary of product characteristic |
| SOC | system organ class |
| THER | date of treatment initiation and end date of reported medication |
| TTO, | Time-to-onset |
