# Supplementary material for: Real-world pharmacovigilance analysis of pralatrexate using the FDA adverse event reporting system database
Source: Front Pharmacol. 2026 Apr 8;17:1773445. doi: 10.3389/fphar.2026.1773445 (PMC13099823; doi:10.3389/fphar.2026.1773445)
Supplement: Supplementary file 2 [file Supplementaryfile1.doc]

"ASC_NTS.DOC" File

for Quarterly Data Extract from the

ADVERSE EVENT REPORTING SYSTEM (AERS)

Revised October 17, 2003

IMPORTANT: The latest changes to the extract and to this document are **bolded**

throughout the text. For a summary of the latest changes, please refer to the

"Revision History" section.

TABLE OF CONTENTS

A. INTRODUCTION

B. FILE DESCRIPTIONS

C. DATA ELEMENT DESCRIPTIONS

D. DATA ELEMENT CONTENTS AND MAXIMUM LENGTHS

E. END NOTES

F. REVISION HISTORY

A. INTRODUCTION

The ASCII data files are '$' delimited; that is, a '$' is used to

separate the data fields. These files can be imported into SAS or into

MS Access or other database program. (Some data files, such as DRUGyyQq

and REACyyQq, will exceed the maximum number of records that can be

imported into spreadsheet programs such as MS Excel.)

In the ASCII format, file names have the format <file-descriptor>yyQq,

where <file-descriptor> is a 4-letter abbreviation for the data

source, 'yy' is a 2-digit identifier for the year, 'Q' is the letter

Q, and 'q' is a 1-digit identifier for the quarter. As an example, the

ASCII demographic file for the 4th quarter of 1997 is represented as

DEMO97Q4. (The set of seven ASCII data files in each extract contains

data for the full quarter covered by the extract.)

B. FILE DESCRIPTIONS

ASCII Data Files:

----------------

1. DEMOyyQq.TXT contains patient demographic and administrative

information, a single record for each event report.

2. DRUGyyQq.TXT contains drug/biologic information for as many

medications as were reported for the event (1 or more per event).

3. REACyyQq.TXT contains all "Medical Dictionary for Regulatory

Activities" (MedDRA) terms coded for the adverse event (1 or more).

For more information on MedDRA, please contact: TRW, VAR1/8A/MSSO,

12011 Sunset Hills Road, Reston, VA 20190-3285, USA; website is

www.meddramsso.com

4. OUTCyyQq.TXT contains patient outcomes for the event (0 or more).

5. RPSRyyQq.TXT contains report sources for event (0 or more).

6. THERyyQq.TXT contains drug therapy start dates and end dates

for the reported drugs (0 or more per drug per event).

7. INDIyyQq.TXT contains all "Medical Dictionary for Regulatory

Activities" (MedDRA) terms coded for the indications for use

(diagnoses) for the reported drugs (0 or more per drug per event).

ASCII Informational Files:

-------------------------

1. ASC_NTS.DOC, which you are reading, shows in some detail the

organization and content of the ASCII data files.

2. STATyyQq.TXT gives null (i.e., no data) counts and frequency

counts for selected fields in the ASCII data sets. (The frequency

counts also include the number of null values; however, the

percentages shown are for non-null values only.)

C. DATA ELEMENT DESCRIPTIONS

1) DEMOGRAPHIC file (DEMOyyQq.TXT)

NAME DESCRIPTION

----------- --------------------------------------------------------------------

ISR Unique number for identifying an AERS report. (See End Note 1.)

This is the primary link field (primary key) between data

files. (example: 3123456)

CASE Number for identifying an AERS case. (Again, see End Note 1.)

A case consists of one or more reports (ISRs). If correctly

linked, a followup report will have the same CASE number

as the initial report. (See End Note 2.)

I_F_COD Code for initial or followup status of report, as reported

by manufacturer. (See table below and End Note 2.)

CODE MEANING_TEXT

---- ------------

I Initial

F Followup

FOLL_SEQ The sequence number of a followup report, as reported by

manufacturer. For initial reports, there will be no data for

this field. (A small number of reports may have "bad" data

for this number, such as alphabetic or special characters.)

(See End Note 2.)

IMAGE Identifier for an AERS report image. Character field consisting of

the ISR number, a dash, and a check digit or letter.

(ex: 3123456-X) See End Note 3.

EVENT_DT Date adverse event occurred or began. (YYYYMMDD format)

MFR_DT Date manufacturer first received initial (or follow-up) information.

(YYYYMMDD format)

FDA_DT Date FDA received report. (YYYYMMDD format)

REPT_COD Code for the type of report submitted. (See table below.)

Also, see End Note 4.

CODE MEANING_TEXT

---- ---------------

EXP Expedited (15-Day)

PER Periodic

DIR Direct

MFR_NUM Manufacturer's unique report identifier. (See Revision

History, Jan - Mar 2002.)

MFR_SNDR Verbatim name of manufacturer sending report. (See End Note 5.)

AGE Numeric value of patient's age at event.

AGE_COD Unit abbreviation for patient's age. (See table below.)

CODE MEANING_TEXT

---- ------------

DEC DECADE

YR YEAR

MON MONTH

WK WEEK

DY DAY

HR HOUR

GNDR_COD Code for patient's sex. (See table below.)

CODE MEANING_TEXT

---- ------------

UNK Unknown

M Male

F Female

NS Not Specified

E_SUB Whether (Y/N) this report was submitted under the

electronic submissions procedure for manufacturers.

WT Numeric value of patient's weight.

WT_COD Unit abbreviation for patient's weight. (See table below.)

CODE MEANING_TEXT

---- ------------

KG Kilograms

LBS Pounds

GMS Grams

REPT_DT Date report was sent. (YYYYMMDD format)

OCCP_COD Abbreviation for initial reporter's type of occupation.

CODE MEANING_TEXT

---- ------------

MD Physician

PH Pharmacist

OT Other health-professional

LW Lawyer

CN Consumer

DEATH_DT Date patient died. (YYYYMMDD format)

TO_MFR Whether or not (Y/N) voluntary reporter also notified

manufacturer (blank for manufacturer reports).

CONFID Whether or not (Y/N) voluntary reporter stated that his

identity should not be disclosed to the product manufac-

turer (blank for manufacturer reports).

2) DRUG file (DRUGyyQq.TXT)

NAME DESCRIPTION

----------- --------------------------------------------------------------------

ISR Number that uniquely identifies an AERS report. Primary link

field between data files.

DRUG_SEQ Unique number for identifying a drug for an ISR. To link to the

THERyyQq.TXT data file, both the ISR number (primary key)

and the DRUG_SEQ number (secondary key) are needed. (For

an explanation of the DRUG_SEQ number, including an

example, please see End Note 6.)

ROLE_COD Code for drug's reported role in event.(See table below.)

CODE MEANING_TEXT

---- ------------

PS Primary Suspect Drug

SS Secondary Suspect Drug

C Concomitant

I Interacting

DRUGNAME Name of medicinal product. If a "Valid Trade Name" is

populated for this ISR, then DRUGNAME = Valid Trade Name;

if not, then DRUGNAME = "Verbatim" name, exactly as entered

on the report. For the great majority of reports, there is

a "Valid Trade Name." (See End Note 5.)

VAL_VBM Code for source of DRUGNAME.(See table below.)

CODE MEANING_TEXT

---- ------------

1 Validated trade name used

2 Verbatim name used

ROUTE The route of drug administration.

DOSE_VBM Verbatim text for dose, frequency, and route, exactly as entered

on report.

DECHAL Dechallenge code, indicating if reaction abated when drug therapy

was stopped. (See table below.)

CODE MEANING_TEXT

---- ------------

Y Positive dechallenge

N Negative dechallenge

U Unknown

D Does not apply

RECHAL Rechallenge code, indicating if reaction recurred when drug

therapy was restarted. (See table below.)

CODE MEANING_TEXT

---- ------------

Y Positive rechallenge

N Negative rechallenge

U Unknown

D Does not apply

LOT_NUM Lot number of the drug.

EXP_DT Expiration date of the drug. (YYYYMMDD format)

NDA_NUM Verbatim NDA number, exactly as reported.

3) REACTION file (REACyyQq.TXT)

NAME DESCRIPTION

----------- --------------------------------------------------------------------

ISR Number that uniquely identifies an AERS report. Primary link field

between data files.

PT "Preferred Term" level medical terminology describing the event,

using the Medical Dictionary for Regulatory Activities (MedDRA).

The order of the terms for a given event does not imply priority.

In other words, the first term listed is not necessarily

considered more significant than the last one listed.

4) OUTCOME file (OUTCyyQq.TXT)

NAME DESCRIPTION

----------- --------------------------------------------------------------------

ISR Number that uniquely identifies an AERS report. Primary link

field between data files.

OUTC_COD Code for a patient outcome. (See table below.)

CODE MEANING_TEXT

---- ------------

DE Death

LT Life-Threatening

HO Hospitalization - Initial or Prolonged

DS Disability

CA Congenital Anomaly

RI Required Intervention to Prevent

Permanent Impairment/Damage

OT Other

5) REPORT SOURCE file (RPSRyyQq.TXT)

NAME DESCRIPTION

----------- --------------------------------------------------------------------

ISR Number that uniquely identifies an AERS report. Primary link

field between data files.

RPSR_COD Code for an initial source of the report. (See table below.)

CODE MEANING_TEXT

---- ------------

FGN Foreign

SDY Study

LIT Literature

CSM Consumer

HP Health Professional

UF User Facility

CR Company Representative

DT Distributor

OTH Other

6) THERAPY dates file (THERyyQq.TXT)

NAME DESCRIPTION

----------- --------------------------------------------------------------------

ISR Number that uniquely identifies an AERS report. Primary link

field between data files.

DRUG_SEQ Drug sequence number for identifying a drug for an ISR. To link to

the DRUGyyQq.TXT data file, both the ISR number (primary

key) and the DRUG_SEQ number (secondary key) are needed.

(For an explanation of the DRUG_SEQ number, including an

example, see End Note 6.)

START_DT A date therapy was started (or re-started) for this drug.

(YYYYMMDD)

END_DT A date therapy was stopped for this drug. (YYYYMMDD)

DUR Numeric value of the duration (length) of therapy

DUR_COD Unit abbreviation for duration of therapy (see table below)

CODE MEANING TEXT

---- ------------

YR Years

MON Months

WK Weeks

DAY Days

HR Hours

MIN Minutes

SEC Seconds

7) INDICATIONS for use file (INDIyyQq.TXT)

NAME DESCRIPTION

----------- --------------------------------------------------------------------

ISR Number that uniquely identifies an AERS report. Primary link

field between data files.

DRUG_SEQ Drug sequence number for identifying a drug for an ISR. To link to

the DRUGyyQq.TXT data file, both the ISR number (primary

key) and the DRUG_SEQ number (secondary key) are needed.

(For an explanation of the DRUG_SEQ number, including an

example, see End Note 6.)

INDI_PT "Preferred Term" level medical terminology describing the Indi-

cation for use, using the Medical Dictionary for Regulatory

Activities (MedDRA).

D. DATA ELEMENT CONTENTS AND MAXIMUM LENGTHS

DATA DATA MAX

ELEMENT CONTENT LENGTH

-------- -------- ------

ISR N (numeric) 10

CASE N 10

I_F_CODE A (alphabetic) 1

FOLL_SEQ AN (alphanumeric) 2

IMAGE AN 12

EVENT_DT N (or D, date) 8

MFR_DT N (or D) 8

FDA_DT N (or D) 8

REPT_COD A 3

MFR_NUM AN 100

MFR_SNDR AN 60

AGE N 7 (including 2 decimal places)

AGE_COD A 3

GNDR_COD A 3

E_SUB A 1

WT N 11 (including 5 decimal places)

WT_COD A 3

REPT_DT N (or D) 8

OCCP_COD A 2

DEATH_DT N (or D) 8

TO_MFR A 1

CONFID A 1

DRUG_SEQ N 10

ROLE_COD A 2

DRUGNAME AN 70

VAL_VBM N 1

ROUTE A 28

DOSE_VBM AN 100

DECHAL N 1

RECHAL N 1

LOT_NUM AN 35

EXP_DT N (or D) 8

NDA_NUM AN 7

PT AN 100

OUTC_COD A 2

RPSR_COD A 3

START_DT N (or D) 8

END_DT N (or D) 8

DUR N 5

DUR_COD A 3

INDI_PT AN 100

E. END NOTES

1 ISR (Demographic and other files). ISR numbers from 11/1/97 to the

present will normally have a length of seven digits. However, because

ISRs were initially planned to be 10 digits, almost 1,300 reports from

November 1997 have 10-digit ISRs. There are about 300 CASE numbers

with 10 digits, for the same reason.

2 CASE, I_F_COD, and FOLL_SEQ (Demographic file). In AERS, a "case"

consists of one or more reports (ISRs). If correctly linked, a followup

report will have the same CASE number as the initial report (but a

different ISR number). It should be kept in mind that the AERS system's determination of what is a follow-up will usually, but not always,

agree with how a manufacturer shows follow-up status on a report.

The I_F_COD code and the FOLL_SEQ number are basically verbatim data

as given by the manufacture; in contrast, the case number is

based on the AERS system's independent determination of what it

considers linked reports. For initial reports, there will be no

FOLL_SEQ number.

3 IMAGE (Demographic file). The Image number is the identifying

number that should be used by anyone requesting sanitized hard copies

of reports through the FOIA. Address: Food and Drug Administration,

Freedom of Information Staff (HFI-35), 5600 Fishers Lane (Rm 12A-16),

Rockville, MD, 20857, USA. Website: www.fda.gov/foi.

4 REPT_COD (Demographic file). Expedited (15-day) and periodic

reports are from manufacturers; "direct" reports are voluntarily

submitted to the FDA by non-manufacturers.

5 MFR_SNDR (Demographic file), DRUGNAME (Drug file), and possibly

other fields. Because of a processing problem in handling the

special characters ampersand ("&"), less-than ("<"), and

greater-than (">"), it has been necessary to remove each such

character, which has been replaced with a period (".").

(Example: MFR_SNDR "Merck Sharp & Dohme" becomes "Merck Sharp . Dohme".)

6 DRUG_SEQ (Drug file and Therapy file). The best way to explain

the DRUG_SEQ (drug sequence number) is with an example. This will also

serve to clarify the relationship between an ISR, the drug(s) reported

for that ISR, and the therapy date(s) reported for the drug(s).

Consider ISR 3078140, received by the FDA on 12/31/97. Like any ISR,

it appears once (and only once) in the Demographic file:

ISR

---

3078140

Four drugs were reported for this ISR: Aricept was reported as

suspect, and Estrogens, Prozac, and Synthroid as concomitant. ISR

3078140 appears four times in the Drug file, with a different DRUG_SEQ

for each drug:

ISR DRUG_SEQ DRUG_VBM

--- -------- --------

3078140 1000157764 Aricept

3078140 1000589550 Estrogens

3078140 1000589552 Prozac( Fluoxetine Hydrochlordie

3078140 1000589553 Synthroid (Levothyroxine Sodium

Dates of therapy for Aricept were reported as "4/97 to 6/13/97",

and "6/20/97 (ongoing)". Since the drug was started, stopped, then

restarted, there are two entries in the Drug Therapy file. In such a

circumstance, the two entries will have the same ISR # and the same

DRUG_SEQ # (see below). No therapy dates were reported for the concomitants;

therefore, they do not appear in the Drug Therapy file, which is

excerpted as follows:

ISR DRUG SEQ # START DT END DT

--- ---------- -------- ------

3078140 1000157764 19970401 19970613

3078140 1000157764 19970620

Another important thing to understand is that the association

between a particular DRUG and a particular DRUG_SEQ # applies only for

a particular ISR. For example, for ISR 3078140 Aricept just by chance

received DRUG_SEQ # 1000157764; should it be reported for other ISRs,

it would receive a different DRUG_SEQ # for each ISR.

F. REVISION HISTORY

June 25, 1999

-------------

1. A Table of Contents was added.

2. A "Revision History" section was added.

July - Sept (3Q), 1999

----------------------

1. In the "Data Element Descriptions" section for "2) DRUG file", a

parenthetical explanation was added to the description of the DRUG_VBM

field, explaining why some software (e.g., SAS) may interpret a small

number of records as "missing" data for this field.

Jan - March (1Q), 2000

----------------------

1. In order to make easier the identification of follow-up reports, three (3)

additional data elements were added to the Demographic file- a Case

number, an initial-or-followup code (I_F_COD), and a sequence number for

follow-ups (FOLL_SEQ). It should be kept in mind that the AERS system's

determination of what is a follow-up will usually, but not always,

agree with how a manufacturer shows follow-up status on a report. The

I_F_COD code and the FOLL_SEQ number are based on the information given

by the manufacturer. On the other hand, the case number is based on the

AERS system's independent determination of what it considers linked

reports.

2. In order to make the drug names easier to use, an important change was

made. Previously, the drug name provided was the "verbatim" name, that is,

the name exactly as entered on the report. Now, the name provided is the

"valid" trade name (based on the AERS drug dictionary), whenever that

field is populated in the database; when valid trade name is NOT populated,

then the verbatim name is provided. (For the 1Q00 data, the valid trade

name is populated 69% of the time.)

3. In order to identify which manufacturer sent a report, a MFR_SNDR

field was added to the Demographic file. This is a "verbatim" name of

the sender, as shown on Form 3500A (item G1). At the same time, the

MFR_VBM field was dropped from the Drug file, because that field is so

rarely populated (3%).

4. The "BRIEF EXPLANATION CONCERNING FOLLOW-UP REPORTS" section was

removed, as it is no longer applicable, given the addition of the CASE,

I_F_COD, and FOLL_SEQ data fields.

Oct - Dec (4Q), 2001

--------------------

1. Electronically submitted reports (e-subs) from manufacturers are

included in the extract for the first time. Previously, because all

manufacturers submitting e-subs were required to also submit a paper

report on the same event, in order to avoid duplication only the paper

report was included in the extract. Starting with this quarter, some

(but not all) manufacturers are now permitted to submit the e-sub alone,

with no duplicating paper report. As a result, e-subs from those

manufacturers are now the only version of the event, and it is

therefore necessary to include e-subs in the extract. Because all

e-subs are now included, there will now be duplicate reports from those manufacturers still required to submit paper reports as backup for the

e-subs. (We have no simple mechanism for including some e-subs in

the extract while excluding others.)

However, such "duplicates" ISRs (multiple reports of the same event)

will normally have the same CASE number (but different ISR numbers).

Users wishing to remove such duplicates can identify a "best ISR"

for the case by use of the CASE and FDA_DT fields- for reports

with the same CASE number, select the latest (most recent) FDA_DT.

For those occasions when both the CASE and FDA_DT fields are the

same, select the report with the higher ISR number. (This procedure

would remove "duplicates" not only in the sense just discussed-

paper and e-sub reports- but also "duplicates" in the sense of

initial and follow-up reports.)

2. During the Oct - Dec 2001 period our MedDRA terminology (the

international medical dictionary) was upgraded to version 4.0.

As a result, many of the PTs ("Preferred Terms") in the extract

have changed.

Jan - Mar (1Q), 2002

--------------------

1. Starting near the end of 2001, and continuing since then, the

MFR_NUM field is populated not just for manufacturer reports but

often for direct reports as well. When populated for direct

reports (REPT_COD='DIR'), the MFR_NUM is actually an internal FDA

system number, and should be ignored by the user of the extract.

Apr - Jun (2Q), 2002

--------------------

1. These additional fields were added to the DEMOyyQq.txt file:

BEST_ISR, E_SUB, WT, WT_COD, REPT_DT, OCCP_COD, DEATH_DT, TO_MFR,

CONFID, and NDA_NUM. Also, the maximum length of the MFR_NUM field was

increased to 100. Please see sections B, C, and D above for details.

2. These additional fields were added to the DRUGyyQq.txt file:

LOT_NUM and EXP_DT. Please see sections B, C, and D above for details.

3. These additional fields were added to the THERyyQq.txt file:

DUR and DUR_COD. Please see sections B, C, and D above for details.

4. A new "txt" data file was added, INDIyyQq.txt, containing the

"Indications for use" for a drug. Please see sections B, C, and D

above for details.

5. Starting with this quarter, the extract includes not just this

quarter’s (02Q2) reports but also reports received during the previous

quarter (02Q1) that were not fully processed into the database in time

for 02Q1’s program “run” on June 17, 2002. (Previously, the extract

included only reports received during the quarter and fully processed

by the date of the run.) From now on, those reports for a quarter that

are not fully processed in time for the run will be included in the

next quarter, so long as they are fully processed in time for the next

quarter’s run. This should “pick up” almost all reports.

Jul - Sep (Q3), 2002

--------------------

1. After last quarter’s (02Q3) extract was made public it was brought to our attention by one of the extract purchasers (thank you!) that some of the ISRs in 2Q02 had already appeared in 02Q1. Additionally, most (approximately 2/3) of those repeating ISRs had an associated CASE number in 02Q2 that was different from the associated CASE number in 02Q1. (For example, ISR 3855605 appears in 02Q1 with CASE # 3769534, and in 02Q2 with CASE # 3805075.) Concerning this situation, there are several points to be made:

a. The phenomenon of ISR numbers repeating in the subsequent quarter (often with a new CASE number) results from certain aspects of AERS processing of incoming reports, combined with certain changes made in the extract processing itself starting in 02Q2. It will likely be present in this and future quarters as well.

b. A repeated ISR number is best considered not as a duplicate but rather as an updated version of the ISR, as it may (or may not) contain updated data. At a minimum, it will often have a new CASE number. Therefore, we recommend that the data associated with the repeated ISR be incorporated into your extract databases, if possible, replacing what is already there for that ISR.

c. As noted above, most of the repeating ISRs had a new CASE # in 02Q2. When this happens, the old CASE # becomes “invalid” and is superceded by the new CASE #. It is recommended that you incorporate the new CASE # into your extract database, replacing the old one. (FYI, in AERS processing, CASE #s may become “invalid” for a number of reasons. Sometimes a CASE # becomes “invalid” because processing has required that a replacement CASE # be created for the same ISR. Other times a CASE # becomes “invalid” because the entire CASE/ISR is no longer considered valid, and there is no replacement CASE #. To allow you to identify “invalid” CASE #s, we hope to include a list of them in the 02Q4 extract.)

d. Some of the repeating ISRs do not involve a new CASE #. As noted above, these also should be considered an update of the existing ISR.

2. The BEST_ISR field has been removed. This field had been added to the Demo file in 02Q2 in order to make it easier to identify which ISR is the “best” (i.e., latest) one for a CASE. However, we are only able to capture “BEST_ISR” status at the date of the program run, whereas what is needed is the status at the end of the quarter. Because of the time lag between end-of-quarter and date of program run, thousands of ISRs that were “best” at the end-of-quarter were no longer “best” at the program run, with the result that thousands of cases in DEMO02Q2 were shown as having no BEST_ISR. Since we can’t fix this problem, we have removed the BEST_ISR field starting in 02Q3. (This problem was brought to our attention by a different extract user- thank you!).

3. Sections C and D were corrected to show that the NDA_NUM field is in DRUGyyQq.txt, rather than DEMOyyQq.txt (as was incorrectly shown in 02Q2).

4. Section D was corrected to show that the Age field in DEMOyyQq.TXT has a max length of 7 digits, including 2 decimals.

5. There was an error in the DRUG02Q2 file: The DECHAL and RECHAL fields were inadvertently reversed in the file, so that DECHAL data appeared in the RECHAL column, and vice-versa. We apologize for this error.

Oct - Dec (Q4), 2002

--------------------

1. An additional file, Invalid_Cases.txt, has been included. This file identifies all Case numbers through the end of 2002 that are classified as “invalid” in AERS. There are more than 18,000 such numbers. While there are various reasons why a Case number becomes classified as invalid, the bottom line is that you will probably want to delete any such Case numbers that may be in your extract database. (It is not known how many of these more than 18,000 Case numbers were included in previous extracts because they were valid at that time, and how many were never included in the extract.) If the file Invalid_Cases.txt proves to be useful to users, and not a source of confusion, we may include it periodically (perhaps in the last quarter of each year).

**Jan – Mar (Q1), 2003**

**--------------------**

**1. There are no changes in this quarter’s formats (compared to the previous quarter). However, AERS’s MedDRA terminology (the international medical dictionary) was upgraded to version 6.0 in May 2003. Starting with the extract for Oct – Dec 2002 (run July 2003), and of course continuing in the current quarter, many of the PTs ("Preferred Terms") in the extract have changed. We forgot to mention this when the Oct – Dec 2002 quarter was made available (July 2003).**
