# Supplementary material for: Real-world pharmacovigilance analysis of pralatrexate using the FDA adverse event reporting system database
Source: Front Pharmacol. 2026 Apr 8;17:1773445. doi: 10.3389/fphar.2026.1773445 (PMC13099823; doi:10.3389/fphar.2026.1773445)
Supplement: Supplementary file 3 [file Supplementaryfile4.pdf]

FREQUENTLY ASKED QUESTIONS (FAQS) FOR THE QUARTERLY DATA EXTRACT (QDE) FROM THE FDA ADVERSE EVENT REPORTING SYSTEM (FAERS)

U.S. FOOD AND DRUG ADMINISTRATION (FDA)
CENTER FOR DRUG EVALUATION AND RESEARCH (CDER)
OFFICE OF SURVEILLANCE AND EPIDEMIOLOGY (OSE)

LAST REVISED: April 2023

1. Why doesn't FAERS include the ISR number? Why did you introduce the new fields PRIMARYID and CASEVERSION into the Quarterly Data Extract?

ANSWER: While the previous Legacy AERS (LAERS) database was Individual Safety Report (ISR) based, the new FAERS database is Case/Version based. In LAERS, a Case consisted of one or more ISRs (Initial and Follow-up reports), and each ISR number represented a separate version of a case. A case could contain multiple ISRs, and the latest ISR represented the most current information about a particular case. (For example, Follow-up 1 would have the most up-to-date information about a case containing both an Initial ISR and a Follow-up 1 ISR).

FAERS will no longer use the ISR concept. Instead, each unique submission of a case received gets a version number (for example, Case# 1234567, Version 1). The first Version we receive (formerly called Initial) will be version 1; the second Version received (formerly called Follow-up 1) will be version 2, and so on. The latest version of a case represents the most current information about a particular Case. As a result, the FAERS QDE output files will always provide the latest, most current Version of a Case available at the time the QDE is run.

2. When I look at a case with multiple versions, I see data only from the latest version. Why is this?

ANSWER: It is possible that a case being updated during a specific quarter to add a subsequent version to the Case will not appear in the QDE output for that particular quarter if it is not yet completed when the QDE output files are prepared. This case will appear in a subsequent output, most likely the following quarter. We do not expect this scenario to affect a significant number of cases each quarter. Another scenario may occur where a version 2 of a case may result in not receiving a version 1 of the case. This would occur if version 1 is both received and updated within a quarter. In that case, only the latest, most current, version 2 will appear in the QDE extract. We expect this scenario to happen infrequently.

In exceptional circumstances, cases may not show up in the extract in which they are expected. The reason is the switch to a Case/Version based system and the version update process. In this situation the cases will show up in the next QDE extract. In each version of a case, users can search for when the initial version of a case was received by FDA by using the <receivedate> XML tag or the INIT_FDA_DATE ASCII field.
The <receiptdate> XML tag or the FDA_RCVD_DATE field in ASCII contains the FDA received date of the individual Case.

3. **Why do we have partial dates now instead of complete dates populated with 01 or 01/01 if partial in a case?**

   ANSWER: While Legacy extracts provided prior to FAERS forced day or month data (i.e., 201202 = 20120201 or 2012 = 20120101) where none was provided, FAERS extracts will provide only the exact data submitted in the case (i.e., 201202 = 201202 or 2012 = 2012). Therefore, partial dates may be present in certain date fields (for example, in XML: <drugstartdate>, <drugenddate> – in ASCII: START_DT, END_DT). We did this to increase data quality, as previously there was a loss of accuracy in some scenarios.

4. **In LAERS, verbatim product information appeared in the ASCII file DOSE_VBM field. Why does it appear much less frequently in the FAERS extract?**

   ANSWER: In the old Legacy extract, we had a single field, DOSE_VBM, containing the verbatim text for dose, frequency, and route (if provided), exactly as it was entered on the report. In the new FAERS extract, four new fields, DOSE_AMT, DOSE_UNIT, DOSE_FREQ, and ROUTE capture the individual data elements for the dose, frequency, and route. The DOSE_VBM field will appear only if data cannot be completely captured in the distinct dose, frequency, and route fields.

5. **Were any fields removed with the FAERS extracts?**

   ANSWER: Yes. We deleted three fields. The QDE will no longer include the following fields due to privacy and other guidelines.

   CONFID
   DEATH_DT
   IMAGE

   Please be sure to note these changes if you have been importing these fields from Legacy AERS into your database.

6. **Why do I sometimes see large numeric values in the ASCII AGE field?**

   ANSWER: In the ASCII AGE field, users may occasionally encounter large numeric age values because some manufacturers submit the patient age in DAYS, even for adult patients. For example, a 30-year-old will be displayed as a 10,950-day-old. Users should note the AGE_UNIT (DY or YR) accompanying the large AGE value and account for ages not listed with the year (YR) age unit.

7. **Why is legacy MedDRA data in all upper case, while new MedDRA data is in sentence (mixed) case?**

   ANSWER: When we transferred LAERS data into the FAERS database, it remained in all upper case (for example, PYREXIA); however, MedDRA terms added into FAERS after September 10, 2012, are in mixed case (for example, Pyrexia).

8. **Does FDA use a drug dictionary?**

   ANSWER: The FAERS system does have a formal dictionary, but it is not publicly available. The primary source of the FDA dictionary is Structured Product Labeling (SPL). You can download data from SPL on the following Web page:

http://www.fda.gov/ForIndustry/DataStandards/StructuredProductLabeling/default.htm

SPL drug names are available to the public at the following link:

http://elist.fda.gov/prpllr/public/query/

We do use other references that are not publicly available (for example, World Health Organization-Drug Dictionary (WHO-DD), NIH's RxNorm, etc.)

You may notice more consistency in the drug and manufacturer data, as we are now relying less on the verbatim data and more on controlled vocabulary terms from our data dictionary.

9. What are the max field lengths specified in the ASC_NTS.doc based upon?

ANSWER: In the ASCII documentation we give the max FAERS field lengths of each field. This allows users to design their databases so that when they do an import, it will not fail because FDA data happens to be larger than the field length in their database. We get these max lengths by looking at the length of the field in the FAERS database that we extract the data from. This was done for the legacy system and we have done it the same way for the new QDE.

10. In the ASCII Demo file, why doesn't FDA use the FOLL_SEQ field anymore?

ANSWER: In the past, we used the I_F_COD and the FOLL_SEQ fields to denote whether an ISR was an Initial or Follow-up and to denote the Follow-up number. FAERS is no longer ISR-based and is now based upon case versioning. We have introduced the CASEVERSION field for tracking the separate versions of a case in our new system. In the ASC_NTS file we have provided the field updates (see table sample below) for reference as QDE users revise their systems to accommodate this change. Under Legacy AERS, FOLL_SEQ was only populated for industry paper reports. In our new FAERS data extract, CASEVERSION is populated both industry paper and industry electronic reports. Voluntary reports from Healthcare providers and consumers do not have follow-up reports.

FOLL_SEQ	NA*	Demo
NA	CASEVERSION*	Demo

11. Why are country codes now showing up as abbreviations instead of full text in the ASCII Demo file (for example, "US" instead of "United States")?

ANSWER: In an effort to standardize the country data, FDA is using ISO standard codes (3166-1) to display Reporter_Country data. Users should use the following links as a reference:

http://estri.ich.org/icsr/ICH_ICSR_Specification_V2-3.pdf
http://www.iso.org/iso/home/standards/country_codes/iso-3166-1_decoding_table.htm

To support this greater standardization, users may need to update their legacy data to the latest codes.

12. How do I determine which drug is the primary suspect product?

ANSWER: In the ASCII extract users should use the field "ROLE_COD" to identify the primary suspect (PS) product as this field explicitly defines this data attribute.

ROLE_COD Code for drug's reported role in event. (See table below.)

CODE	MEANING_TEXT
PS	Primary Suspect Drug
SS	Secondary Suspect Drug
C	Concomitant
I	Interacting

13. Why is <occurcounty> tag added to the XML extract and OCCR_COUNTRY field added to the ASCII extract?

ANSWER: The <occur country> XML tag and the OCCR_COUNTRY ASCII field will help you to distinguish between domestic and foreign cases.

14. Why are some of the statistical files Null Counts columns headers modified from how they were being displayed in the past?

ANSWER: The changes to the Stat files were done to clarify the description of the Null Count data being provided in the Indication, Therapy, and Reaction files. In some cases we have added a column to show what is provided to help clarify exactly what is missing.

15. Why are some of the Report Source data counts lower in FAERS?

ANSWER: Please remember that FAERS only gives the latest data of a case. In the past, if versions contained multiple report sources all would be captured. Under FAERS, only the latest version report sources are captured.

16. I am seeing isolated cases where the version does not seem correct. Am I missing data?

ANSWER: We have had to correct some isolated cases and this may present itself in the extract. However, you will always be getting all of the data that we have.

17. I've noticed in your statistics report of the first FAERS extract (2012 Q4), the percentage of Death cases has increased from approximately 12% to 19%. Why is this occurring?

ANSWER: Our new system has created a backlog of cases and to mitigate this we have prioritized inputting of all Death cases.
http://www.fda.gov/Drugs/DrugSafety/ucm358264.htm

18. I've noticed that some Direct cases have a version other than one. This did not occur in the legacy data. What is the reason for this?

ANSWER: In most cases, this has to do with new procedures to deal with cases where compounding may be an issue.

19. Why is Null not the predominant choice in the Rechallange statistics report?

ANSWER: This has been corrected starting with the 2015 Q4 extract.
NOTE: There will be no data cleanup or reposting of corrected data.

20. I've noticed a change in the percentage of non-Null values in the TO_MFR field – why is this?

ANSWER: After investigation, it was determined that the default settings for this data element did not match what appeared on the MedWatch paper forms. As a result the data profile for this element in the 2012Q4 through 2013Q4 extracts does not match the data profile in the previous LAERS extracts. However, this issue is now corrected and as a result from the 2014Q1 extract forward this data element will appear similar to what it did in the LAERS extracts.
NOTE: There will be no data cleanup or reposting of corrected data.

21. I've noticed that the REPORTER_COUNTRY missing count is now showing "0" in the Demo Stat file but in the LAERS Stat file it was never "0" – why is this?

ANSWER: We are now providing the term "Country not specified" when no reporter country is provided in the case. Therefore, the stat file will always show "0".

22. I noticed that you are using "Country Not Specified" in the Reporter Country field but are not using "Not Specified" for other fields that are unknown – why is this?

ANSWER: This is being done to ensure DTD validation occurs. Both Reporter Country and Qualification tags cannot be blank or this will cause an error.

23. In the NDA field, I noticed that the numbers "999999" are sometimes present – why is this?

ANSWER: For many years, the FDA has populated this field with "999999" where the suspect drug is an Over-the-Counter (OTC) product.

24. I've noted that in the first FAERS extract (2012 Q4), case numbers 7334857, 8791047, 9300033 have an entry in the Drug file that has no drug name - why is this?

ANSWER: These records represent data errors where drug names were incorrectly erased. You can disregard these rows. Additionally, you can disregard information related to these rows in any other file.

25. How do I know what version of MedDRA that an individual extract is based on?

ANSWER: The MedDRA version is indicated in the <reactionmeddraversionpt> tag of the XML file.

26. In the first FAERS XML extract (2012 Q4), the <reporttype> tag was not filled in 100% of the time as it was in AERS – why is this?

A.1.4	reporttype	Type of report;<br>Code List:<br>1= Spontaneous<br>2= Report from Study<br>3= Other<br>4= Not available to sender (unknown)

ANSWER: This is now corrected.

27. The first FAERS XML extract (2012 Q4), contained invalid MedDRA terms in the indications field – why is this?

ANSWER: This has been corrected in the 2013 Q1 extract; all extracts will contain only valid MedDRA terms.

28. I had some trouble processing the first FAERS extract (2012 Q4) because some of the data fields contained special characters.

ANSWER: Some companies have submitted data that contains characters that have caused problems with imports in some systems. These characters may be non-printable characters in the ASCII data sets or even characters outside the wider UTF-8 Unicode standard.

Quarterly Data Extracts now only include printable ASCII characters, line feed (ANSI #10), and carriage return (ANSI #13). If an unapproved character is present, it will be replaced with a "?".

29. I am seeing a few duplicate records between your 2012 Q4 and 2013 Q1 extracts, case and version number are identical in these records. What should I do with these?

ANSWER: The latest record will contain the most complete information and that case should be used.

30. I have found cases with no Primary Suspect (ROLE_COD=PS) product within the DRUG ASCII file of the QDE – why is this?

ANSWER: All cases should contain one Primary Suspect product; however, cases with no Primary Suspect (PS) have been identified within the extract. Users should designate the first drug listed as the Primary Suspect product if one is missing.

31. How does FAERS link different versions of a case together and are there any exceptions that I have to think about when creating a database or querying the data?

ANSWER: Voluntary (aka Direct) reports are not linked by the FAERS system, each report is a separate case.

Mandatory reports by companies often have follow-up reports with additional information. Manufactures supply a manufacturer control number (MCN) with each report they submit. FDA checks this number at the beginning of data entry or during the electronic submission process and if the MCN matches a

case in the system that version is assigned the case number of the case that it matches. If there is no match a new case is started.

There are two special cases worth mentioning:

A. In the case of paper reports, manufacturers can arbitrarily choose an MCN number and it is possible that two manufacturers choose the same number. In general, FDA resolves this issue and will give each the correct case numbers but you will see MCN numbers that occur in more than case occasionally because of this.
B. As stated the paper MCN number is arbitrarily chosen by the manufacturer; however, in the world of electronic submission a special format is used which includes the manufacturer name to ensure all MCN's are unique. When a manufacturer changes from paper to electronic submission the MCN number will change. Again FDA takes care of this and will assign the correct case number but you will occasionally see a case with non-matching MCN numbers.

Programmers should use case versions to understand how different reports (case versions) are linked. FDA needs to create these links with MCN numbers but there are a few exceptions where MCN numbers match multiple cases or do not match within a case. Generally, programmers will not need to use MCN numbers but if they chose to do so they should be aware of the exceptions.

32. Does the FDA provide support for software used to import the QDE data for analysis? After importing the QDE data, why are some of the variables/fields blank?

ANSWER: The FDA does not provide technical support for individual software packages used for QDE data consumption. It should be noted that most of this data is voluntary and much of it is not filled in. It may help to review the QDE statistical reports that show the rates that different fields are filled in and compare it with what is imported.

33. In the 2014Q2 extract the number of cases outside the Q2 extract period seems larger than normal – why is this?

ANSWER: It was discovered that a small subset of follow-up cases in each extract starting with 2012Q4 were missed during the extraction process. We identified these cases and included them in the 2014Q2 extract.

34. The Demographics (DEMO) file contains the date the adverse event occurred or began (EVENT_DT). Will the FDA provide this information in the XML file?

ANSWER: Starting with the 2014Q3 extract, we are providing the event start date at the case level in our XML file just as we do in the ASCII File. Since a tag for Case Event Date does not exist in the E2B standard this data, when available, will be in the B.5.1 <narrativeincludeclinical> tag and displayed as follows:

"CASE EVENT DATE: Event Date"

Date Format YYYYMMDD or YYYYMM or YYYY

NOTE: This tag does NOT include Case Narrative.

Event date is the date that the adverse event occurred as supplied by the reporter. If the reporter does not supply the date it will not appear in the extract.

35. In the 2014Q3 extract new fields and tags were added, will the FDA provide these tags/fields for the previous FAERS extracts?

ANSWER: There are no current plans to provide this data. Additional case data can be ascertained through the FOIA request process.

36. In the 2014Q3 extract, the XML tag for <narrativeincludeclinical> containing the CASE EVENT DATE was added. In a case where several drugs and reactions are provided what information can be used to calculate "time-to-onset"? Also, should CASE EVENT DATE apply to all reactions OR only to the first term highlighted by the reporter?

ANSWER: Please consider the following information when using the data provided in the <narrativeincludeclinical> XML tag:

A. There is no XML tag for "Onset Date" at the case level in E2B; therefore, the **<narrtiveincludeclinical>** tag was repurposed (see Question 34).
B. The Event Onset Date can come from either paper reports or E2B submissions as follows:
   i. In paper reports, Event Onset Date is provided at the case level and is defined as the actual or best estimate of the date of first onset of the adverse event.
   ii. In E2B submissions, we take the first Event Start Date listed in the case as the case level Event Onset Date. This date is provided in the QDE XML extract at the case level and should not necessarily be associated with a particular suspect product in the case.
C. We do not believe that the possible temporal relationships between Therapy Start Date and Event Onset Date prove causality with the products in the case (i.e., a correlation between the Primary Suspect or Secondary Suspect drugs and the Event(s) listed in the case).

The Case Event Date could apply to ALL Reactions listed in the case since this date is being provided only at the Case Level.

NOTE: Providing this data in the XML extract in this format ensures that we are being compliant with the Freedom of Information Act release of Personally Identifiable Information (PII) policy and allows for consistency between both ASCII and XML extracts.

37. Could you provide more information about the new field for Drug Recur Action (DRUG_REC_ACT) in the Event file populated with the Reaction/Event information?

A. What are the meanings of the Rechallenge values as they relate to this field?

ANSWER: The values for the DRUG_REC_ACT field are the Reactions, if any, which reoccurred when a suspect product(s) was reintroduced.

NOTE: Paper Cases marked with a value of Rechallenge = Y will not display reaction terms in the new DRUG_REC_ACT field as MedWatch forms do not associate any specific reactions to this data element. However, E2B cases marked with a value of Rechallenge = Y may display reactions in the DRUG_REC_ACT field if it is populated with a valid Preferred Term (PT) or Lower Level Term (LLT).

B. When there are multiple reactions in the report, are they all given the same value?

RESPONSE: That is correct – there is no weight/seriousness given to any of the reactions.

C. If there are multiple drugs listed in the case, can the list of reactions provided in this field can be associated with any of the Suspect drugs?

RESPONSE: A QDE user may conclude that at least one of the Suspect drugs was reintroduced to cause the reaction(s) to reoccur.

38. In trying to import the DRUG ASCII file into Microsoft Excel, I get the following error message: "File not loaded completely." What should I do to import the file into Excel?

RESPONSE: In Microsoft Excel, the maximum worksheet size is 1,048,576 rows by 16384 columns. If/when the ASCII file rows exceed the maximum worksheet limitation, open the ASCII file in a text editor (e.g., Notepad, Microsoft Word). Save the ASCII file as several smaller files that conform to this row limitation, and then import the smaller files into Microsoft Excel.

39. I would like to understand how the relationship between the seven ASCII files is structured. Is there an "Entity Relationship Diagram" (aka ERD) available?

RESPONSE: The ASC_NTS document was updated and SECTION B now details how the relationship between the files is structured. In the same section, you may also find the Entity Relationship Diagram (ERD).

40. I have noticed that the number of cases containing Report Source information has been declining in recent extracts. Why is this happening?

RESPONSE: The Report Source file (RPSRyyQq.TXT) contains information from Block G.3 of the Paper 3500A (Mandatory) Form submitted to the FDA. Per the recent mandate, all drug manufacturers are expected to report to the FDA electronically. The electronic standard does not capture Report Source as the Paper 3500A (Mandatory) Form did in Block G.3 and therefore it is not provided.

41. I noticed the counts for Dechallenge in this Extract (2016 Q3) is different than past Quarterly Data Extracts? Also, what are the meanings of the Dechallenge values?

ANSWER: There is no XML tag for "Dechallenge" (Abated) in E2B; therefore, FDA has implemented specific logic that derives this information from the Action Taken and Event Outcome E2B tags.
NOTE: There will be no data cleanup or reposting of corrected data.

42. I noticed that in some 2016 and 2017 quarters the total number of records in the physical file does not exactly match the total number of records from the pdf report on the data. Why is this?

ANSWER: We are aware that the total number of records in the current data files do not match the total number of records stated in the reports that support the data files beginning Q1, 2016. In 2016, FDA determined that a small number of industry reports on blood and tissue products should not be included in the data extracts because they were submitted to the FDA voluntarily from companies before it was mandatory to submit them. Therefore, these reports were removed from the data files in 2016. The record counts in the tables were not updated, which resulted in a small discrepancy (< 0.05%) between the record counts and the count of reports in the data files from the first quarter of 2014 until the third quarter of 2017

43. I noticed that in some of the quarters the total number of records in the physical file does not exactly match the total number of records from the pdf report on the data. Why is this?

ANSWER: We are aware that the total number of records in the current data files do not match the number of records stated in the pdf reports that support the data files. This discrepancy exists beginning from the fourth quarter of 2012 until the third quarter of 2017.
In 2016, FDA determined that a small number of industry reports on blood and tissue products should not be included in the data extracts because they were submitted to the FDA voluntarily from companies before it was mandatory to submit them. Therefore, these reports were removed from the data files in 2016. The record counts in the pdf reports were not updated, which resulted in a small discrepancy (< 0.05%) between the number of physical records present in the extract and the counts expressed in the supporting pdf reports.

44. If I compare only records in the latest QDE quarter to the same latest Public Dashboard quarter, will the counts be exactly same?

ANSWER: They may not be; if FDA receives an initial record and its follow up in the same quarter, the QDE will only show the latest version, while the Public Dashboard will show both records on its home page that displays report version counts. Note also that the QDE has a greater number of fields than the Public Dashboard and the QDE includes concomitant drugs while the Public Dashboard only has reported suspect drugs. Also, note that the FAERS Public Dashboard is comprised of two distinct sections. The first section is the home page, which is a summary of all case report versions received by the FDA. This section allows a high-level representation of case version counts by year, by quarter, and month. The second section allows users to search, analyze and export case level data for only the latest version of each case. This section is useful for analysis based on drug or event-based searches. These sections combined present a holistic view of the FAERS data in a user-friendly manner.

45. As of 2019 Quarter one there is a new text file that lists deleted files. What is this used for?

ANSWER: FDA or Manufacturers may delete cases for various reasons including combining cases. Depending on the date of the extract and the date when the

delete occurred these may show up in QDE. We are supplying these deleted cases to bring QDE and our public dashboard into greater alignment.

46. In Q1 2020, the MedWatch Online Form has added new reporter occupation values that can be reported. How are these new reporter occupation values accounted for in the QDE output files?

ANSWER: The MedWatch Online form now allows new reporter occupation values to be reported. These will be mapped to existing e2b standard occupation values in both ascii (rpsr_cod column) and xml (A.2.1.4 <qualification> tag) output files as shown below.

- Other Health Professional, Nurse Practitioner, Dentist, Physician Assistant, Biomedical Engineer: All these new values are mapped to "Healthcare Professional"
- Non Health Professional, Risk Manager, Administrator/Supervisor, Third Party servicer: All these new values are mapped to "Consumer"

47. Starting in 2021 Q4 extract it seems that DUR and DUR_CODE values have increased. Why is this?

ANSWER: FAERS modernization revised the retrieval of the DUR and DUR_COD to include both direct and mandatory cases.

48. In the Indication .pdf report, why does the "Total Count of Drugs" not equal the sum of "Total Count of Indications" and "Missing Count"?

ANSWER: There is not a one-to-one correlation between drugs and indications. Some drugs may have more than one indication reported and some drugs may have no indication reported.

The updated column definitions are provided below.

Data Type – "Indication"
Total Count of Drugs – Total count of drug records in the file.
Total Count of Indications – Total count of indication records reported
Missing Count – Total count of drug rows where indication was not reported.

49. In the .pdf Drug file, the percentages for Routes have changed. Why is this?

ANSWER: In the .pdf Drug file, the Route table's null count was corrected to display the actual count of the null records. As a result, the percentages of many routes were impacted.

50. In the .pdf Drug file, the percentages for VAL_VBM have changed. Why is this?

ANSWER: In the .pdf Drug file, the Drug table's null count was corrected to display the actual count of the null records. As a result, the percentages of many VAL_VBM were impacted.

51. The total number of records in 2021 Q4 is lower than expected. Why is this?

ANSWER: This was caused by producing the extract later than expected due to system modernization. As a result, the missing records from 2021 Q4 will be published in 2022 Q1.

52. We used to see negative ages in unborn patients. These cases are now showing zero instead of a negative number. Why is this?

ANSWER: While data is sometimes captured in reports for adverse effects during pregnancy, the new FAERS system only captures the patient data with a positive age beginning at patient age zero which is inclusive of Neonates. You can further search on cases with the age of zero to investigate potential impacts to a foetus during pregnancy. Additionally, MedDRA terms (e.g., LLT Exposure during pregnancy) can also be used to query FAERS data and determine exposure of a neonate or foetus during pregnancy.

53. Why am I seeing more than one drug record for a given sequence number (i.e., drug_seq_no) for some of the products listed in the ASCII Drug file for Direct reports?

ANSWER: In the Drug file, there may be more than one drug record for each product listed in a report received from Healthcare Professionals and Consumers (aka. Direct reports). Reports can have multiple dosing, route, or frequency information for a given product. Starting with 2021Q4, QDE provides all of this information for a given product. Therefore, you should adjust your import program to allow for one or more rows per drug sequence number (i.e., Primary_id_n and drug_seq_no) to correctly import this data into your database.

54. Why am I seeing the same data in the 'DRUGNAME' and 'PROD_AI' fields for some product types?

ANSWER: A recent dictionary update has caused changes in the 'DRUGNAME' field of QDE. In the case of herbals, this causes the 'DRUGNAME' and 'PROD_AI' to have the same value that was formally expressed only in the 'PROD_AI' field. FDA is studying this change and considering the impact of any further changes on all parts of the dictionary, FAERS database and Quarterly Data Extract and Public FAERS dashboard.
